# Endophytic Fungi Associated with Seaweeds as Potential Producers of Antimicrobial Compounds

**DOI:** 10.1007/s10126-026-10626-1

**Published:** 2026-05-13

**Authors:** Maria da Luz Calado, Débora Santos, Patrícia Susano, Susete Pintéus, Alice Martins, Joana Silva, Miguel A. M. Oliveira, Celso Alves, Rui Pedrosa, Patrick G. Murray, Katie Shiels, Maria Jorge Campos

**Affiliations:** 1 MARE - Marine and Environmental Sciences Centre & ARNET - Aquatic Research Network, ESTM, Polytechnic University of Leiria, Edifício CETEMARES, Avenida do Porto de Pesca, Peniche, 2520- 630 Portugal; 2 LIFE - Health and Biosciences Research Institute, Technological University of the Shannon, Limerick, V94EC5T Ireland

**Keywords:** Marine endophytic fungi, Seaweed mycobiota diversity, Antimicrobial activity, Mechanisms of action, Fungal extracts

## Abstract

The urgent need for new molecules capable of targeting multidrug-resistant microorganisms has redirected bioprospecting efforts toward less explored marine environments and their associated communities, such as marine mycobiota. This study aimed to evaluate the antimicrobial potential of endophytic fungi isolated from various seaweed species and infer the mechanisms of action and chemical profile of most potent fungal extracts. Simultaneously, it intended to contribute to a better understanding of ecological requirements of each fungal species. Thirty-one ascomycetous fungi were recovered from the seaweed hosts, with a higher representation of the genera *Aspergillus* and *Penicillium*. Only two fungal species revealed notable antimicrobial abilities: *Penicillium rubens* synthesised extracts with pronounced antibacterial effects against *Escherichia coli*,* Pseudomonas aeruginosa* and *Staphylococcus aureus*, while extracts of *Penicillium brevicompactum* inhibited the growth of *Candida albicans*. Although similar activities have been described in a few previous studies, this is the first report of a markedly inhibitory effect against a pathogenic yeast demonstrated by *P. brevicompactum.* Moreover, the mechanisms of action underlying these activities were explored for the first time in this study. Specifically, the antimicrobial activities demonstrated by fungal extracts were likely mediated by membrane potential imbalance and DNA damage, associated with the presence of L-norvaline and formylphosphonate. Despite the overall consistency of the results, slight to pronounced differences were also observed in the antibacterial activities and mechanisms of action of the extracts from *P. rubens* strains. These findings support the hypothesis that marine fungal species possess, and express distinct metabolic profiles shaped by micro- and/or macro-environmental factors.

**Supplementary Information:**

The online version contains supplementary material available at 10.1007/s10126-026-10626-1.

## Introduction

The recent emergence of multidrug resistant pathogens as a consequence of an overuse and misuse of antibiotics has been contributing to a higher occurrence and lethality of infectious diseases (Silber et al. 2016). This alarming tendency reinforces the importance and urgency of discovering novel classes of antibiotics with different modes-of-action (Fischbach and Walsh 2009).

Most of the efforts to overcome this need are focused on the bioprospection of natural products, specially those produced by microorganisms, given their recognition as major producers of antimicrobial compounds (Newman and Cragg 2020). According to Fischbach and Walsh (Fischbach and Walsh 2009), more than two-thirds of antibiotics are natural-based products or a result of their semisynthetic derivates.

Marine microorganisms and, specifically, marine fungi are receiving ever-increasing attention from the scientific community due to their ability to produce chemically and structurally novel and diverse compounds with marked bioactivities (Bugni and Ireland 2004; Rateb and Ebel 2011; Silber et al. 2016; Sridhar 2017; Carroll et al. 2019).

Marine fungi represent an ecological group of fungi that, according to the most recent and consensual definition, includes all the species that are exclusively associated with marine ecosystems - marine fungi *sensu stricto* - but also species that demonstrated to be physiologically well-adapted and metabolically and functionally active in those ecosystems - osmotolerant/halotolerant fungi (Pang et al. 2016; Overy et al. 2019; Amend et al. 2019). Accordingly, 2254 species are currently accepted as marine fungi (19/01/2026; https://www.marinefungi.org/).

Marine fungi assume a symbiotic, parasitic, or saprotrophic lifestyle, occurring associated with different marine hosts/substrates from the intertidal to deep oceanic habitats (Amend et al. 2019). The harsher and stressful ecological conditions of marine environments (e.g., nutrient limitation, temperature and salinity fluctuations, sunlight exposure in intertidal habitats, and high hydrostatic pressure in deeper areas) have been hypothesised to be the main responsible factors for triggering an adaptative response by these fungi and, concretely, for inducing the biosynthesis of bioactive secondary metabolites (Ebel 2012; Debbab et al. 2012; Reich and Labes 2017).

The marine endophytic fungi, in particular, have been recently recognised as an emerging and prolific source of compounds with biotechnological application (Zhang et al. 2016; Sarasan et al. 2017; Rashmi et al. 2018; El-Bondkly et al. 2021).

Marine endophytic fungi occur within seaweeds, seagrasses, and/or different intertidal halophytes, inhabiting inter- and/or intracellularly their internal tissues, in highly diverse communities. Even though the interactions established between endophytic fungi and their hosts are not fully understood, most of the relationships have been demonstrated to be beneficial for both partners (Suryanarayanan et al. 2012; Zhang et al. 2016; Sarasan et al. 2017; El-Bondkly et al. 2021). Apparently, the hosts seem to provide the nutrient supply and protection against predators and external threats to their fungal partners and, in return, the fungi synthesise secondary metabolites that promote the growth and contribute to the acclimatization and adaptation of their hosts to environmental stresses, competitors and diseases (Debbab et al. 2012). The antagonistic effect of marine fungal secondary metabolites against pathogenic bacteria is believed to occur through the inhibition of bacterial growth or biofilm formation, or by reducing the virulence of the strains (Zhuravleva et al. 2022).

Focusing specifically on the ecological niches occupied by seaweeds, some of the most stressful factors may be the *(i)* extended exposure to sunlight and consequent desiccation, *(ii)* variable salt concentrations, *(iii)* nutrient limitation, and *(iv)* biotic colonization (pathogenic virus, bacteria, fungi and algae) (Zhang et al. 2016; Rashmi et al. 2018; Vallet et al. 2018; Sarasan et al. 2020; Sahoo et al. 2021). Seaweeds are among the most relevant hosts for endophytic fungal producers of bioactive compounds (Bugni and Ireland 2004; Rateb and Ebel 2011; Suryanarayanan 2012; Singh et al. 2015; Zhang et al. 2016; Sarasan et al. 2017; Ogaki et al. 2019), including those with antimicrobial activities (Tarman et al. 2011; Flewelling et al. 2013b, a; Furbino et al. 2014; Wong et al. 2015; Gnavi et al. 2016; Vallet et al. 2018; Handayani et al. 2019). Most of the compounds produced by fungal endophytes associated with seaweeds are polyketides, terpenoids, alkaloids, steroids, and nitrogen-containing compounds (Debbab et al. 2012; Mousa and Raizada 2013; Singh et al. 2015; Zhang et al. 2016; Sarasan et al. 2017).

Nevertheless, a very limited number of inventories of the microbiota associated with these hosts have been performed (Zuccaro et al. 2003; Loque et al. 2010; Suryanarayanan et al. 2010; Jones et al. 2012; Flewelling et al. 2015; Wong et al. 2015; Gnavi et al. 2017; Sarasan et al. 2017; Ogaki et al. 2019; Cooper and Walker 2022; Poli et al. 2022). According to Flewelling et al. (2015), less than 1% of the worldwide known species of seaweeds have been surveyed for the presence of fungal endophytes. In Portugal, only a few studies focused on the fungal communities associated with seaweeds (Sridhar et al. 2012; Gonçalves et al. 2019, 2020; Vicente et al. 2021). This knowledge gap compromises any attempt to predict the fungal species that might occur on a certain seaweed species and geographic region. The current knowledge suggests that endophytic communities associated with seaweeds are dominated by non-host-specific species but also include less common or rare species (Suryanarayanan et al. 2010; Godinho et al. 2013; Furbino et al. 2014). Some of these endophytic fungal species might exhibit some degree of tissue- (different parts/organs) and/or host-specificity (Vallet et al. 2018; Wainwright et al. 2019).

Thus, the present study aims to address this gap in knowledge based on two key premises: (1) intensifying efforts in exploring diverse hosts across different geographic regions will provide valuable insights into the distribution patterns and ecological requirements of each fungal species (Sridhar et al. 2012); (2) distinct strains of the same fungal species, associated with different hosts and/or geographical locations, are likely to exhibit varied metabolic responses to environmental constraints (Suryanarayanan et al. 2010; Venkatachalam et al. 2015; Amend et al. 2019; Wainwright et al. 2019; Marchese et al. 2020; Ghoran et al. 2022).

Specifically, this study mainly intends to assess the culturable endophytic fungal species associated with some of the most dominant or frequent seaweed species inhabiting the Western coast of Portugal and evaluate their putative ability to biosynthesise compounds with antimicrobial properties. Moreover, the cell damage mechanisms behind the antimicrobial activities of the compounds will also be evaluated.

## Materials and Methods

### Collection of Seaweeds and Isolation of Fungi

Fresh and apparently healthy thalli of sixteen species of seaweeds were randomly collected at three sandy beaches in the Peniche peninsula (Gambôa: 39.3651˚N, 9.3728˚W; Portinho da Areia Norte: 39.3691˚N, 9.3779˚W; Baleal, Praia dos Barcos: 39.3766˚N, 9.3402˚W) from June to December 2018: two green (*Codium* sp. and *Ulva compressa*), six red (*Bornetia secundiflora*, *Corallina elongata*, *Gelidium pulchellum*, *Gelidium sesquipedale*, *Plocamium cartilagineum*, and an identified Rhodophyta), and eight brown (*Bifurcaria bifurcata*, *Cladostephus spongiosus*, *Cystoseira tamariscifolia*, *Fucus spiralis*, *Padina pavonica*, *Saccorhiza polyschides*, *Sargassum* cf. *muticum*, and *Sargassum vulgare*). *Sargassum muticum* and *Fucus guiryi* were additionally collected at a more distant sandy beach (Leça da Palmeira: 41.1883˚N, 8.7070˚W), to obtain a more representative collection of the endophytic fungi inhabiting the seaweeds of the West coast of Portugal.

All samples were promptly transported to the laboratory and carefully rinsed with sterile seawater to eliminate surface debris, sediments, and transient fungal propagules. The samples were subsequently subjected to a surface sterilization process to eliminate the epiphytes, involving sequential immersion in sterile seawater, 70% ethyl alcohol (for 5 s), followed by two rinses in sterile seawater (Kjer et al. 2010; Sarasan et al. 2017). Each sample was aseptically cut into segments of approximately 1 cm² and directly plated onto Malt Extract Agar (MEA) and Corn Meal Agar (CMA), supplemented with chloramphenicol. The use of a nutrient-rich (MEA) and a -poor medium (CMA) intended to maximize the isolation of species with different nutritional requirements and growth rates (Gnavi et al. 2017; Garzoli et al. 2018; Fan et al. 2019; Lee et al. 2019a). The imprint method recommended by Zuccaro et al. (2003) and Zhang et al. (2016) was performed with a several treated segments of each algal sample to confirm the effectiveness of the surface sterilization process. The inoculated plates were incubated at 28 °C, in the dark, and checked daily for the presence of new hyphae grown out of the cut edges of the seaweed fragments. Each fungus was sequentially transferred to new plates with Potato Dextrose Agar medium and incubated under the same conditions until pure cultures were obtained. All the isolates were maintained in a slower active growth at 4 °C and cryopreserved.

### Molecular Identification of Fungal Isolates

Considering the high number of fungi isolated from all the seaweed samples, only different morphotypes were chosen for molecular identification and further procedures.

Briefly, the identification method involved the extraction of genomic DNA by a phenol-chloroform protocol adapted from Liu et al. (2000), followed by PCR amplification and sequencing of internal transcribed spacers 1 and 2, and intervening 5.8 S region of the nuclear ribosomal DNA (ITS), using the primer pair IT5 and ITS4 (White et al. 1990). Complementary genetic markers (ribosomal DNA and protein-coding genes) were also amplified for specific groups, as the ITS region is not effective in distinguishing different species within those groups. The following primers pairs were used in this study: NS1/NS4 for partial nuclear ribosomal DNA small subunit (18 S) (White et al. 1990); LROR/LR5 for partial nuclear ribosomal DNA large subunit (28 S) (Stielow et al. 2015); EF1-983/EF1-2218r, EF1-728/EF2 for partial translation elongation factor 1-alpha (tef1-α) gene (Grum-Grzhimaylo et al. 2013; Heo et al. 2018); CALDF1/CALDR1, CMD5/CMD6 for partial calmodulin (cal) gene (Lawrence et al. 2013; Samson et al. 2014; Visagie et al. 2014; Siqueira et al. 2016; Woudenberg et al. 2017); RPB2-5 F/RPB2-7CR for RNA polymerase II (RPB2) gene (Woudenberg et al. 2013; Grum-Grzhimaylo et al. 2013; Visagie et al. 2014; Siqueira et al. 2016); ACT512F/ACT783R for partial actin (act) gene (Bensch et al. 2012, 2015); T1/Bt2b and Bt2a/Bt2b for partial β-tubulin (tub) gene (Visagie et al. 2014; Yilmaz et al. 2014; Siqueira et al. 2016; Heo et al. 2018; Gonçalves et al. 2020). The conditions of polymerase chain reactions (PCR) and the purification process applied to amplicons are described in Calado et al. (2021).

The obtained sequences were compared with those deposited in the GenBank database and identified to the lowest possible taxonomic level. Sequences were then deposited in the GenBank database (Accession numbers available in the Supplementary Table 1).

A multilocus phylogenetic analysis was also performed with a few fungal isolates to clarify or confirm their phylogenetic placement, species delimitation, and their relationships with other members of the same clade. This approach was carried out using genes with stronger phylogenetic signals. Prior to this, phylogenetic analyses for individual *loci* were performed to assess the concordance of the resulting tree topologies.

These analyses involved the alignment of sequences of different genes/regions from isolates and best BLAST matches, and the construction of a phylogenetic tree based on maximum Likelihood (ML) criteria. ML analyses were based on the best-fit nucleotide substitution model, selected according to the lowest Akaike Information Criterion (AIC) value. Alignment gaps were handled as partial deletions, applying a 95% site coverage cut-off. ML trees were inferred using the Nearest-Neighbour-Interchange (NNI) algorithm as the heuristic search method, with an initial neighbour-joining (NJ) tree automatically generated by the software. The relative robustness of the clades was evaluated by performing 1000 bootstrap replicates.

**Fig. 1:**
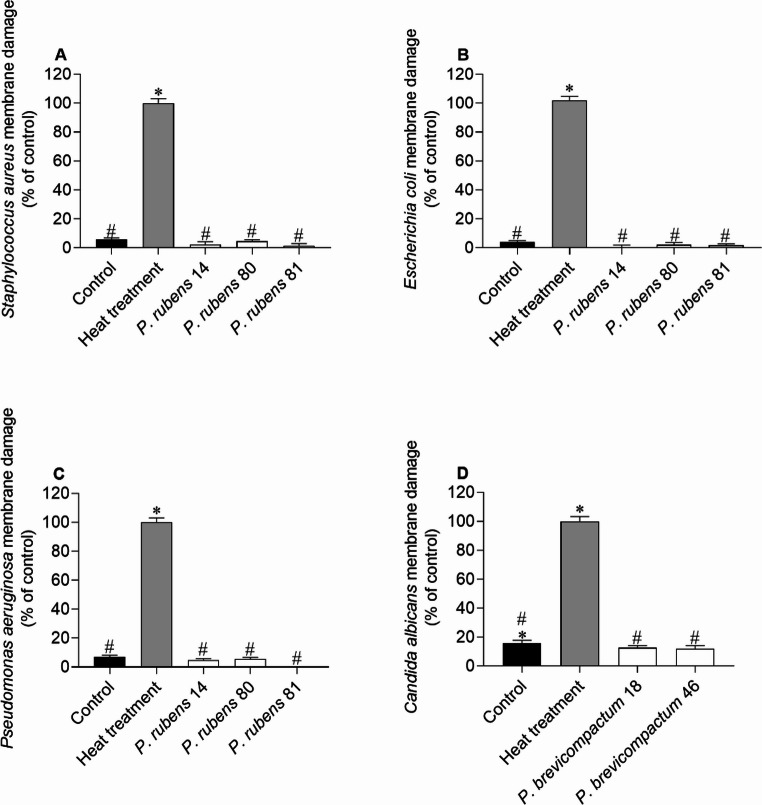
*Staphylococcus aureus* **(A)**, *Escherichia coli* **(B)**, *Pseudomonas aeruginosa* **(C)** and *Candida albicans* **(D)** membrane integrity when exposed to extracts (IC_50_ values), tagged with Sytox Green probe. DMSO was used as negative control (untreated cells), and bacterial cells exposed to a heat treatment (10 min, 100 °C) were used as a positive control of membrane damage. The values correspond to the mean ± SEM of three independent experiments. Symbols represent significant differences (One-Way ANOVA, Dunnett’s test; *p* < 0.05) when compared to the control (*) and heat treatment (#)

**Fig. 2:**
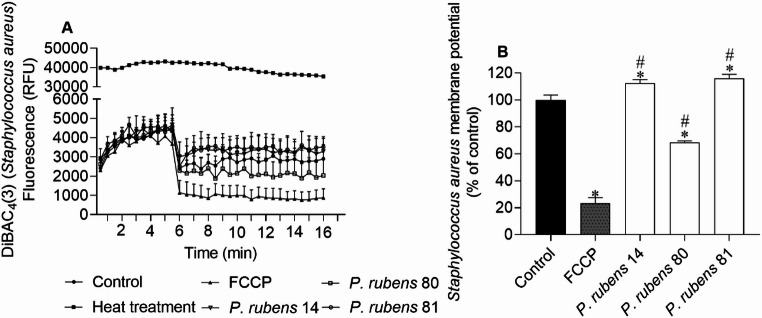
*Staphylococcus aureus* membrane potential when exposed to extracts (IC_50_ values of 4.7, 13.2, and 5.0 µg/mL for extracts of isolates 14, 80 and 81, respectively), labelled with DiBAC_4_(3) probe; **(A)** 30 s interval readings for 10 min; **(B)** after 10 min. DMSO was used as a negative control and FCCP (100 µM) as a positive control of membrane potential alterations. The values correspond to the mean ± SEM of three independent experiments. Symbols represent significant differences (One-Way ANOVA, Dunnett’s test; *p* < 0.05) when compared to the control (*) and FCCP (#)

The datasets that revealed similarity amongst tree topologies were then concatenated, and a multilocus phylogenetic analysis was further performed using the same ML criteria. The resulting trees are provided as supplementary material (Figs. 1 and 2). All the analyses were carried out in MEGA11 software.

### Small-scale Fermentation Assay and Extraction of Secondary Metabolites

A small-scale fermentation procedure was carried out for all the isolated strains, as well as nine additional fungal strains previously isolated from the same sampling period, locations and seaweed hosts (Calado et al. 2021; Oliveira et al. 2025), namely, *Aspergillus fructus*, *Emericellopsis maritima*, *Exophiala mesophila*, *Microascus croci*, *Penicillium toxicarium*, *Purpureocillium lilacinum*, *Stemphylium gracilariae* and *Talaromyces pinophilus*.

This procedure involved the growth of each strain in Wickerham´s medium broth at 28 °C under static, dark conditions for a variable period (17 to 47 days), depending on fungal growth. The criterion was to stop the fermentation process when the fungus showed a clear reduction in growth rate, which may indicate a shift to secondary metabolism. Then, the potential secondary metabolites retained in the mycelial biomass and/or released into the fermentation broth were extracted using ethyl acetate as solvent (Calado et al. 2021). Combined organic extracts were concentrated *in vacuo* and kept at −20 °C until further analyses. The extracts were resuspended in DMSO at 20 mg/mL for antimicrobial assays.

### Evaluation of Antimicrobial Activities of Fungal Extracts

The antimicrobial properties of the fungal extracts were tested on five pathogenic microorganisms obtained from the German Collection of Microorganisms and Cell Cultures (DSMZ): Gram-negative bacteria *Escherichia coli* (DSM 1103), *Klebsiella pneumoniae* (DSM 16358) and *Pseudomonas aeruginosa* (DSM 1117), Gram-positive bacteria *Staphylococcus aureus* (DSM 1104), and the fungus *Candida albicans* (DSM 1386). The procedure involved an initial growth of bacteria and fungi in appropriate broth media, followed by adjustment of the microbial density to an OD_600_ of 0.125. Subsequently, the microorganisms were cultured in 96-well plates containing each fungal extract at a final concentration of 200 µg/mL, and incubated at 37 °C. The results were obtained during the exponential growth phase of each microorganism. Oxytetracycline (Sigma-Aldrich, St. Louis, MO, USA) was used as a positive control for *E. coli*, *K. pneumoniae*, *P. aeruginosa* and *S. aureus*, and amphotericin B (Sigma-Aldrich, St. Louis, MO, USA) for *C. albicans*. The results were expressed as percentage of control (with DMSO (Sigma-Aldrich, St. Louis, MO, USA) instead of sample). A dose–response analysis was carried out for the most active extracts (> 50% inhibition) using doses ranging from 10 to 200 µg/mL to determine the IC_50_ values.

### Study of Antimicrobial Mechanisms of Action

In order to identify the possible mechanisms that might underlie the inhibitory effects, further assays were carried out on the extracts that showed the highest inhibitory activity (> 50% at 200 µg/mL).

#### Membrane Damage Assay

Membrane damaging potential was performed as described by Pinteus et al. (2020). Briefly, a freshly overnight grown microorganism’s liquid culture was centrifuged (2000 x g, 5 min) and resuspended in 0.85% of sterile saline solution at OD_600_ = 0.5. The extracts were added at the concentration of IC_50_ value and incubated for 4 h at 37 °C. DMSO (instead of the extracts) was used as a negative control. A positive control was obtained by subjecting the suspension to a thermal treatment (100 °C, 10 min) to induce complete membrane permeability. Sterility controls were prepared with extracts and sterile saline solution (without microorganisms). After incubation, all suspensions were transferred to a black microplate and incubated with 2 µM Sytox Green (10 min, at 37 °C, in the dark). Then, the fluorescence of the DNA-bound dye was measured using a fluorescence microplate reader (Synergy H1 Multi-Mode Microplate Reader, BioTek^®^ Instruments, Winooski, USA) with excitation at 535 nm and emission at 595 nm. The membrane damage was determined as a percentage of the positive control.

#### Membrane Potential Analysis

The membrane potential variation assay was performed as described by Clementi et al. (2014), combining two fluorescent dyes, namely the potentiometric dye bis-(1,3-dibutyl barbituric acid) trimethine oxonol (DiBAC_4_(3)) (Thermo Fisher Scientific, Waltham, MA, USA) with the DNA-staining dye propidium iodide (PI) (Sigma Aldrich, Darmstadt, Germany). Firstly, an overnight-grown culture of the microorganism was centrifuged twice (1200 x g, 10 min), resuspended in Phosphate-Buffered Saline (PBS) at 1.5 McFarland, and supplemented with 0.25% of a 1 M glucose stock solution. Forty-five microliters of DiBAC_4_(3) (25 µM) and 90 µL of PI (500 µg/mL) were added to the microbial solution, which was then distributed through 96-well black plates (99 µL/well) and incubated at 37 °C, 1 h, in the dark. Blanks were prepared equally but with PBS instead of the microbial solution. A positive control for total membrane permeabilization was obtained with heat-treated bacteria (100 °C, 10 min). A solution of carbonyl 4-(trifluoromethoxy) phenylhydrazone (FCCP) and amphotericin B were used as positive controls for hyperpolarization for the bacteria and fungus, respectively. After incubation, 1 µL of extract was quickly added at IC_50_ and the fluorescence was read (λ excitation: 490 nm, λ emission: 516 nm for DIBAC; and λ excitation: 535 nm, λ emission: 617 nm for PI) with 30 s intervals for 10 min on a fluorescence microplate reader (Synergy H1 Multi-Mode Microplate Reader, BioTek^®^ Instruments, Winooski, USA). The results are presented in DiBAC4(3) relative fluorescence units (RFU).

#### DNA Damaging Potential

The DNA damaging potential was evaluated by the methodology previously described by Hu et al. (2017). Briefly, 5 µL of plasmid DNA (pGADT7–7987 bp) at a final concentration of 100 ng/mL was mixed with 2 µL of the extracts (200 µg/mL) and 13 µL of ultrapure water. The reaction mixtures were incubated for 1 h at 37 °C before loading onto a 0.8% agarose gel containing 10% GelStarTM. Three microliters of Gene Ruler 1 Kb DNA Ladder (ThermoFisher Scientific, Waltham, MA, USA) were also loaded onto the gel. Electrophoresis was then performed for 45 min under 85 V. DMSO and ciprofloxacin (Sigma-Aldrich, St. Louis, MO, USA) (1 mM) were used as negative and positive controls, respectively. The results were visualized in a Gel DOC E3 Imager (Biorad, Califórnia, EUA).

### Chemical Screening by LC-Q-TOF MS

The fungal extracts that demonstrated high antimicrobial activity were further chemically characterised.

#### Sample Extraction

In brief, 1 g of the extracts was weighed into a 50 mL conical tube, and 2 mL of 60:40 acetonitrile (ACN): methanol (MeOH) was added. The samples were sonicated for 10 min in an ultrasonic bath at room temperature. Then, samples were incubated at 37 °C to allow the bioactive constituents to be extracted. Following incubation, the supernatant was removed from the 50 mL conical tube, transferred into a 2 mL microtube, and stored at −20 °C until analysis.

#### LC-Q-TOF MS Analysis

The extracts were analysed using an Agilent HPLC connected to a Q-TOF mass spectrometer (Agilent 6520) to obtain the chemical profile of samples.

Chromatographic conditions were optimised based on preliminary experiments (data not shown). The samples were separated using a reversed-phase (RP) chromatographic method on an Agilent Poroshell 120 EC-C_18_ (4.6 × 150 mm, 2.7 μm) HPLC column. During analysis, the sample tray temperature was maintained at 4 °C, with an injection volume of 5 µL and a column temperature of 34 °C.

The mobile phase flow rate was kept at 0.6 mL min⁻¹ throughout the gradient. A 5% acidified acetonitrile and 95% acetonitrile, both with 0.1% (v/v) of formic acid (Sigma-Aldrich) were used as eluent A and eluent B, respectively. The following gradient profile was established: 0–3 min, 4% B; 3–8 min, 4–20% B; 8–15 min, 20–70% B; 15–25 min, 70–90% B; 25–40 min, 90–100% B; 40–50 min, 100% B. A post-time of 5 min was allocated to balance the initial conditions for the next analysis.

A dual electrospray ionization (ESI) source was employed in positive ion mode. The mass spectrometric parameters were set as follows: drying gas temperature, 325 °C; drying gas flow rate, 13 L min⁻¹; nebulizer pressure, 40 psi; capillary voltage, 4500 V; fragmentor voltage, 175 V; and skimmer voltage, 65 V. Data were collected over an *m/z* range of 60–1100 at a scan rate of 2.4 spectra s⁻¹.

#### Data Processing

To disclose the chemical profile of samples, two distinct and specific approaches were employed: (1) unknown-known compounds; and (2) unknown-unknown analysis. In both approaches, data on all organic compounds were extracted from each sample by the Mass Hunter analytical software, taking into consideration appropriate quality parameters of the detected analyte, i.e. isotopic ratio, mass/charge variations, various adducts and transition.

The unknown-known compounds approach involved checking the list of obtained compounds against recently updated chemical compound libraries, such as the Agilent METLIN secondary metabolites library. The results were arranged in the data quality ranking and the top 20 compounds from over a thousand discovered entries were reported in the supplementary material.

The unknown-unknown analysis involved organizing the list of obtained compounds according to the ranking of signal intensity, which reflects the abundance of the compounds in the sample. The top 20 analytes, if not found in the standard Agilent libraries of compounds, were searched for in an online database of chemical compounds (https://metlin-nl.scripps.edu/landing_page.php?pgcontent=mainPage).

### Statistical Analyses

A one-way analysis of variance (ANOVA) followed by Dunnett’s multiple comparison test was performed to evaluate significant differences from the control. Data normality and variance homogeneity were assessed using the Shapiro–Wilk and Levene’s tests, respectively. If the assumptions for ANOVA were not fulfilled, a non-parametric Kruskal–Wallis test followed by Dunn’s multiple comparison was used. IC_50_ values were calculated using GraphPad v9.3.1 software, applying the formula Y = 100/(1 + 10^(x-log(IC_50_))). All computations and final graphical presentations were created with GraphPad v9.3.1 (GraphPad Software, La Jolla, CA, USA). Data represent results from three independent experiments performed in triplicate, and are shown as the standard error of the mean (SEM), with significance set at *p* < 0.05.

## Results

### Culturable Marine Endophytic Fungi Associated with Different Seaweed Hosts

The molecular identification process revealed thirty-one ascomycetous fungal taxa (Table 1), most of which were identified to the species level (87%).

**Table 1:** Endophytic fungi recovered from algal samples, molecularly identified by comparison of sequences of one or more barcode genes with those published on Genbank database. The identification of fungal species marked with an asterisk resulted from a multilocus phylogenetic analysis

Identified fungal taxa (isolate code)	Seaweed host species	Sequenced genes	BLAST results	
			Discriminatory locus: Cover; Identity (%)	BLAST best hits (NCBI accession nº)
***Dothideomycetes***				
***Capnodiales***				
*Cladosporium allicinum* (10)	*Fucus guiryi* ^(L1)^	ITS, act	act: 98%; 94%	*Cladosporium allicinum* (EF101354)
*Cladosporium ramotenellum* (41)	*Bornetia secundiflora*^(G4)^	ITS, act	act: 100%; 100%	*Cladosporium ramotenellum* (MG680539)
*Cladosporium austrohemisphaericum* (33)	Rhodophyta ^(B3)^	ITS, act	act: 97%; 98%	*Cladosporium austrohemisphaericum* (KT600578)
***Pleosporales***				
Periconia byssoides* (43)	*Padina pavonica* ^(G4)^	ITS, 18 S, 28 S, tef1-α	ITS: 100%; 99%	*Periconia byssoides* (MK370654)
*Alternaria* sp. 1 (section Infectoriae; 70)	*Cladostephus spongiosus* ^(G5)^	ITS	ITS: 100%; 100%	*Alternaria heterospora* (MH863292)
*Alternaria* sp. 2 (section Ulocladioides; 72)	*Cladostephus spongiosus* ^(G5)^	ITS	ITS: 100%; 100%	*Alternaria infectoria* (MT548683)
*Stemphylium lycopersici* (49)	*Cladostephus spongiosus* ^(G5)^	ITS, cal	cal: 99%; 100%	*Stemphylium lycopersici* (MK895976)
*Stemphylium vesicarium* (42)	*Sargassum vulgare* ^(G4)^	ITS, cal	cal: 100%; 100%	*Stemphylium vesicarium* (MN410932
***Sordariomycetes***				
***Hypocreales***				
*Sarocladium* sp. (77)	*Plocamium cartilagineum* ^(G5)^	ITS, 28 S	ITS: 98%; 99%	*Sarocladium strictum* (OW982735)
*Emericellopsis maritima* (31)	Rhodophyta ^(B3)^	ITS, tef1-α, tub	tef1-α: 98%; 100%	*Emericellopsis maritima* (FJ238393)
*Emericellopsis maritima* (76)	*Cladostephus spongiosus* ^(G5)^	ITS, tef1-α, tub	tef1-α: 97%; 100%	*Emericellopsis maritima* (FJ238393)
***Microascales***				
Yunnania carbonaria* (66)	*Corallina elongata* ^(G5)^	ITS, 28 S, tef1-α, tub	tef1-α: 100%; 100%	*Yunnania carbonaria* (KX924045)
***Lulworthiales***				
*Lindra obtusa* (32)	Rhodophyta ^(B3)^	ITS, 18 S, 28 S	ITS: 100%; 100%	*Lindra obtusa* (LC146744)
***Xylariales***				
*Apiospora marii* (68)	*Cladostephus spongiosus* ^(G5)^	ITS, tub	tub: 99%; 100%	*Apiospora marii* (MH384417)
***Eurotiomycetes***				
***Chaetothyriales***				
*Exophiala mesophila* (47)	*Padina pavonica* ^(G4)^	ITS	ITS: 96%; 97%	*Exophiala mesophila* (KP003829)
***Eurotiales***				
***Aspergillus***				
section Aspergillus				
*Aspergillus pseudoglaucus* (12)	*Sargassum muticum* ^(L1)^	ITS, cal	cal: 99%; 100%	*Aspergillus pseudoglaucus* (MN031425)
section Candidi				
*Aspergillus subalbidus* (75)	*Cladostephus spongiosus* ^(G5)^	ITS, tub, cal	cal: 99%; 99%	*Aspergillus subalbidus* (LT908035)
section Fumigati				
*Aspergillus fumigatus* (28)	*Ulva compressa* ^(B3)^	ITS, cal	cal: 100%; 100%	*Aspergillus fumigatus* (MG991521)
section Restricti				
*Aspergillus conicus* (34)	Rhodophyta ^(B3)^	ITS, cal	cal: 98%; 100%	*Aspergillus conicus* (HE578091)
section Versicolores				
*Aspergillus austroafricanus* (84)	*Fucus spiralis* ^(B6)^	ITS, cal, tub	cal: 99%; 100%	*Aspergillus austroafricanus* (LC558332)
*Aspergillus protuberus* (3)	*Gelidium pulchellum* ^(B2)^	ITS, cal	cal: 100%; 99%	*Aspergillus protuberus* (LT594411)
*Aspergillus protuberus* (9)	*Fucus guiryi* ^(L1)^	ITS, cal	cal: 100%; 100%	*Aspergillus protuberus* (LN898788)
*Aspergillus protuberus* (23)	*Codium* sp. ^(P3)^	ITS, tub, RPB2	tub: 100%; 100%	*Aspergillus protuberus* (JN853968)
*Aspergillus protuberus* (82)	*Saccorhiza polyschides* ^(B6)^	tub, RPB2	tub: 99%; 100%	*Aspergillus protuberus* (MF575005)
*Aspergillus sydowii* (25)	*Sargassum* cf. *muticum* ^(P3)^	ITS, cal	cal: 100%; 100%	*Aspergillus sydowii* (LN898812)
***Penicillium***				
section Aspergilloides				
*Penicillium glabrum* (7)	*Sargassum muticum* ^(L1)^	ITS, tub	tub: 99%; 100%	*Penicillium glabrum* (FJ004409)
*Penicillium spinulosum* (11)	*Fucus guiryi* ^(L1)^	ITS, cal	cal: 100%; 100%	*Penicillium spinulosum* (DQ911126)
section Brevicompacta				
*Penicillium brevicompactum* (18)	*Fucus guiryi* ^(L1)^	ITS	ITS: 100%; 100%	*Penicillium brevicompactum* (MT558924)
*Penicillium brevicompactum* (46)	*Sargassum vulgare* ^(G4)^	ITS, RPB2	ITS: 100%; 100%	*Penicillium brevicompactum* (KX664324)
section Canescentia				
*Penicillium antarcticum* (87)	*Fucus spiralis* ^(B6)^	ITS, tub, RPB2	tub: 100%; 100%	*Penicillium antarcticum* (MK519554)
section Chrysogena				
*Penicillium rubens* (14)	*Bifurcaria bifurcata* ^(B2)^	ITS, tub	tub: 100%; 100%	*Penicillium rubens* (MN369582)
*Penicillium rubens* (80)	*Saccorhiza polyschides* ^(B6)^	ITS, tub, cal, RPB2	tub: 100%; 100%	*Penicillium rubens* (MN395867)
*Penicillium rubens* (81)	*Saccorhiza polyschides* ^(B6)^	ITS, tub, cal	tub: 100%; 100%	*Penicillium rubens* (MN395867)
*Penicillium rubens* (95)	*Gelidium sesquipedale* ^(B6)^	ITS, tub, RPB2	tub: 100%; 100%	*Penicillium rubens* (MN395867)
section Citrina				
*Penicillium citrinum* (45)	*Cladostephus spongiosus* ^(G4)^	tub	tub: 100%; 100%	*Penicillium citrinum* (MN882791)
*Penicillium citrinum* (78)	*Cladostephus spongiosus* ^(G4)^	ITS, tub	tub: 100%; 100%	*Penicillium citrinum* (MN418435)
*Penicillium citrinum* (94)	*Plocamium cartilagineum* ^(B6)^	ITS, tub	tub: 100%; 100%	*Penicillium citrinum* (MN882791)
***Talaromyces***				
*Talaromyces* sp. 1 (section Trachyspermi; 4)	*Cystoseira tamariscifolia* ^(B2)^	ITS, tub	tub: 100%; 100%	*Talaromyces assiutensis* (KJ865720)
***Leotiomycetes***				
*Botrytis cinerea* (*Botryotinia fuckeliana*; 27)	*Bifurcaria bifurcata* ^(B3)^	ITS	ITS: 100%; 100%	*Botrytis cinerea* (MN077161)
***Pezizomycotina incertae sedis***				
*Asteromyces cruciatus* (6)	*Padina pavonica* ^(G2)^	ITS	ITS: 99%; 100%	*Asteromyces cruciatus* (MK432716)
*Asteromyces cruciatus* (65)	*Corallina elongata* ^(G5)^	ITS	ITS: 98%; 100%	*Asteromyces cruciatus* (MK432716)
*Asteromyces cruciatus* (69)	*Cladostephus spongiosus* ^(G5)^	ITS	ITS: 99%; 100%	*Asteromyces cruciatus* (MK432716)
***Saccharomycetes***				
*Geotrichum candidum* (73)	*Cladostephus spongiosus* ^(G5)^	ITS	ITS: 99%; 99%	*Geotrichum candidum* (MK381259)

Location: ^(G)^ Gambôa; ^(P)^ Portinho da Areia Norte; ^(B)^ Baleal; ^(L)^ Leça da Palmeira

Collection date: ^(1)^ May 2018; ^(2)^ June 2018;^(3)^ August 2018; ^(4)^ September 2018; ^(5)^ October 2018; ^(6)^ December 2018

Even though most fungal strains were identified based on one or two genes, the identification of *Periconia byssoides* (isolate 23) and of scopulariopsis-like taxon *Yunnania carbonaria* (isolate 66) required the construction of ML phylogenetic trees based on single and multiple gene sequence alignments. The ML analyses were concordant in the phylogenetic placement of the isolates, with a high bootstrap support (data not shown). On the other hand, the identification of *Alternaria* sp. 1 (isolate 70) and *Alternaria* sp. 2 (isolate 72) was only possible to the section level.

Taking the results obtained from all seaweeds together, there is a clear dominance of three taxonomic classes, i.e., Eurotideomycetes, Dothideomycetes and Sordariomycetes, which encompassed 48%, 26% and 16% of the recovered fungal taxa, respectively. *Aspergillus* and *Penicillium* were the most representative and frequent genera, given the higher number of different species and strains isolated from the surveyed seaweeds.

Twenty-five fungal taxa were recovered from a single seaweed species, while six fungal species were detected in two or more seaweed hosts, in the same or different sampling periods and locations (Table 1). *Penicillium rubens* was found exclusively at Baleal beach, associated with one brown seaweed in June, and with another brown and one red seaweed species in December; *Asteromyces cruciatus* was identified solely at Gambôa beach, as an endophyte of one brown seaweed in June, and of another brown and one red seaweed species in October; *Emericellopsis maritima* was isolated from a red seaweed species in August, and from a brown seaweed species collected at a different geographical location, in October; *Penicillium brevicompactum* was recovered from two brown seaweed species, collected at two geographically distant locations and different periods; *Penicillium citrinum* was identified in one brown seaweed species collected at Gambôa beach, and later in one red seaweed from Baleal beach.

Even though different groups of seaweeds have not been equally sampled, the results suggested an apparent higher fungal richness in brown and red seaweed species. Specifically, the brown seaweed species harboured between one to nine fungal taxa, the red seaweed species presented one to four fungal taxa, and the two green seaweed species revealed one fungus each.

### Inhibitory Effects of Fungal Extracts Against Pathogenic Microorganisms

Among all tested extracts, only those synthesised by two *Penicillium* species were able to inhibit microorganisms’ growth in more than 50%, and thus subjected to a dose response analysis.

The extract of isolate 80 of *P. rubens* exhibited the highest inhibition against *P. aeruginosa* and *E. coli*, with IC_50_ values of 1.2 (0.7–2.0.7.0) µg/mL and 3.2 (1.6–5.7) µg/mL, respectively. Similarly, extracts of isolates 81 and 14 of the same species also demonstrated significant antibacterial activity; isolate 81 showed IC_50_ values of 4.9 µg/mL against *P. aeruginosa* and 6.1 µg/mL against *E. coli*, and isolate 14 exhibited IC_50_ values of 104.0 µg/mL and 8.9 µg/mL against *P. aeruginosa* and *E. coli*, respectively. The extracts obtained from isolates 14, 81 and 80 also demonstrated a high inhibitory activity against *S. aureus*, with IC_50_ values of 4.7 µg/mL (4.0–5.4.0.4), 5.0 µg/mL (3.9–6.4) and 13.2 µg/mL (11.3–15.6), respectively. None of the extracts inhibited the growth of *K. pneumoniae*.

While the above-mentioned extracts exhibited antibacterial activities, the extracts obtained from isolates 18 and 46 of *P. brevicompactum* presented antifungal properties, inhibiting the growth of *C. albicans*, with IC_50_ values of 19.5 (14.7–25.8) µg/mL and 47.8 (38.0–54.7) µg/mL, respectively.

### Potential Antimicrobial Mechanisms of Action

The most active extracts were further analysed for their impact on microorganisms’ membrane and on DNA integrity. Thus, isolates 14, 80 and 81 of *P. rubens* were analysed against *S. aureus*, *E. coli* and *P. aeruginosa*, while isolates 18 and 46 of *P. brevicompactum* were analysed against *C. albicans*.

#### Membrane Damage Evaluation

The effects of the most active extracts on the membrane integrity of *S. aureus*,* E. coli*,* P. aeruginosa* and *C. albicans*, are shown in Fig. 1.

Data presented in Fig. 1 suggest that the extracts did not promote significant membrane damage to the target microorganisms, since no significant differences were found when comparing any of the extracts with the untreated control.

#### Membrane Potential Evaluation

**Fig. 3:**
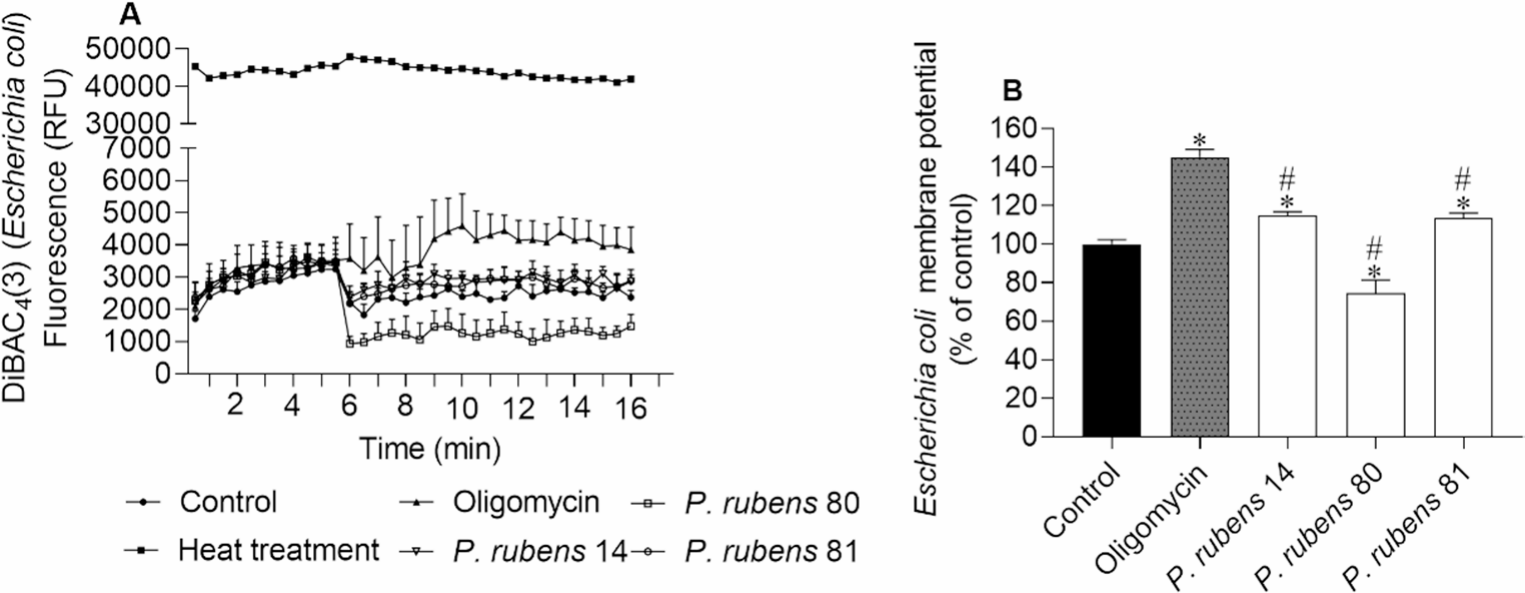
*Escherichia coli* membrane potential when exposed to extracts (IC_50_ values of 8.9, 3.2, and 6.1 µg/mL for extracts of isolates 14, 80 and 81, respectively), labelled with DiBAC_4_(3) probe; **(A)** 30 s interval readings for 10 min; **(B)** after 10 min. DMSO was used as a negative control and oligomycin (0.4 mg/mL) as a positive control of membrane potential alterations. The values correspond to the mean ± SEM of three independent experiments. Symbols represent significant differences (One-Way ANOVA, Dunnett’s test; *p* < 0.05) when compared to the control (*) and oligomycin (#)

**Fig. 4:**
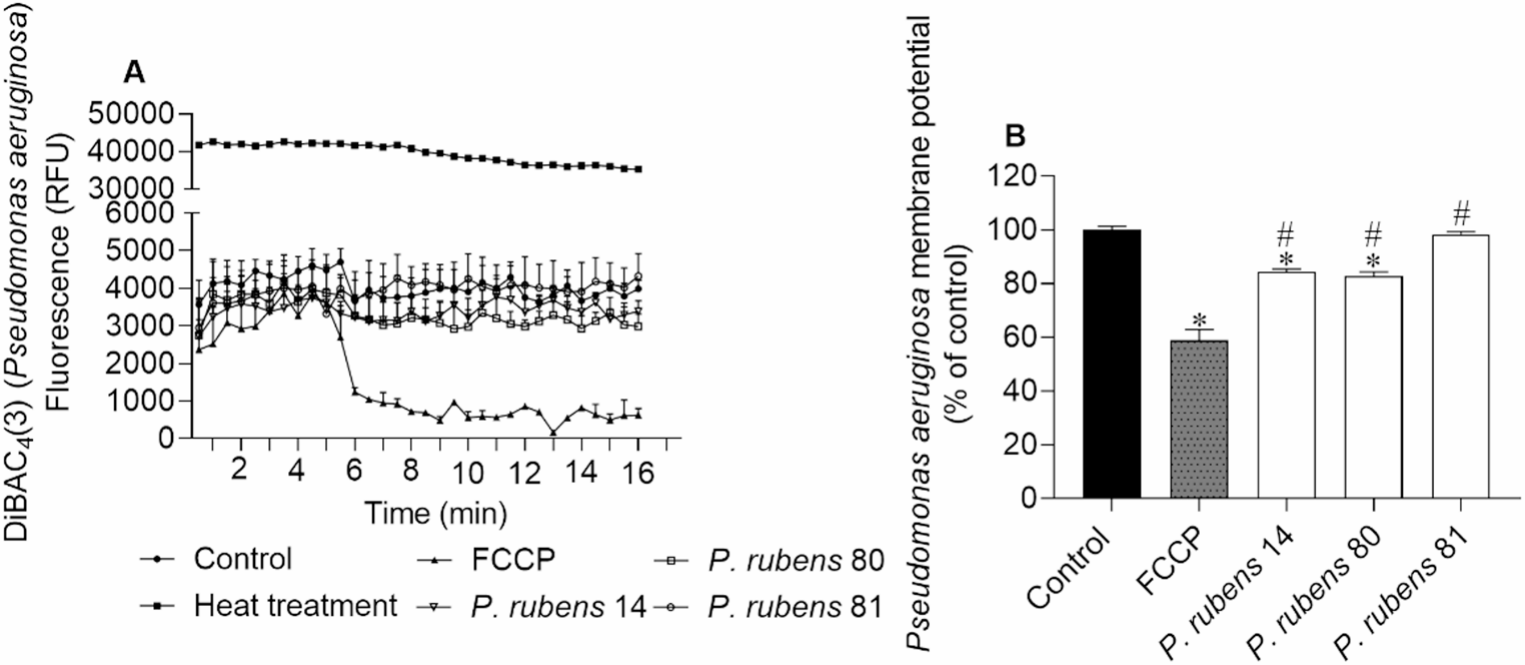
*Pseudomonas aeruginosa* membrane potential when exposed to extracts (IC_50_ values of 104, 1.2, and 4.9 µg/mL for extracts of isolates 14, 80 and 81, respectively), labelled with DiBAC_4_(3) probe; **(A)** 30 s interval readings for 10 min; **(B)** after 10 min. DMSO was used as a negative control and FCCP (1 mM) as a positive control of membrane potential alterations. The values correspond to the mean ± SEM of three independent experiments. Symbols represent significant differences (One-Way ANOVA, Dunnett’s test; *p* < 0.05) when compared to the control (*) and FCCP (#)

**Fig. 5:**
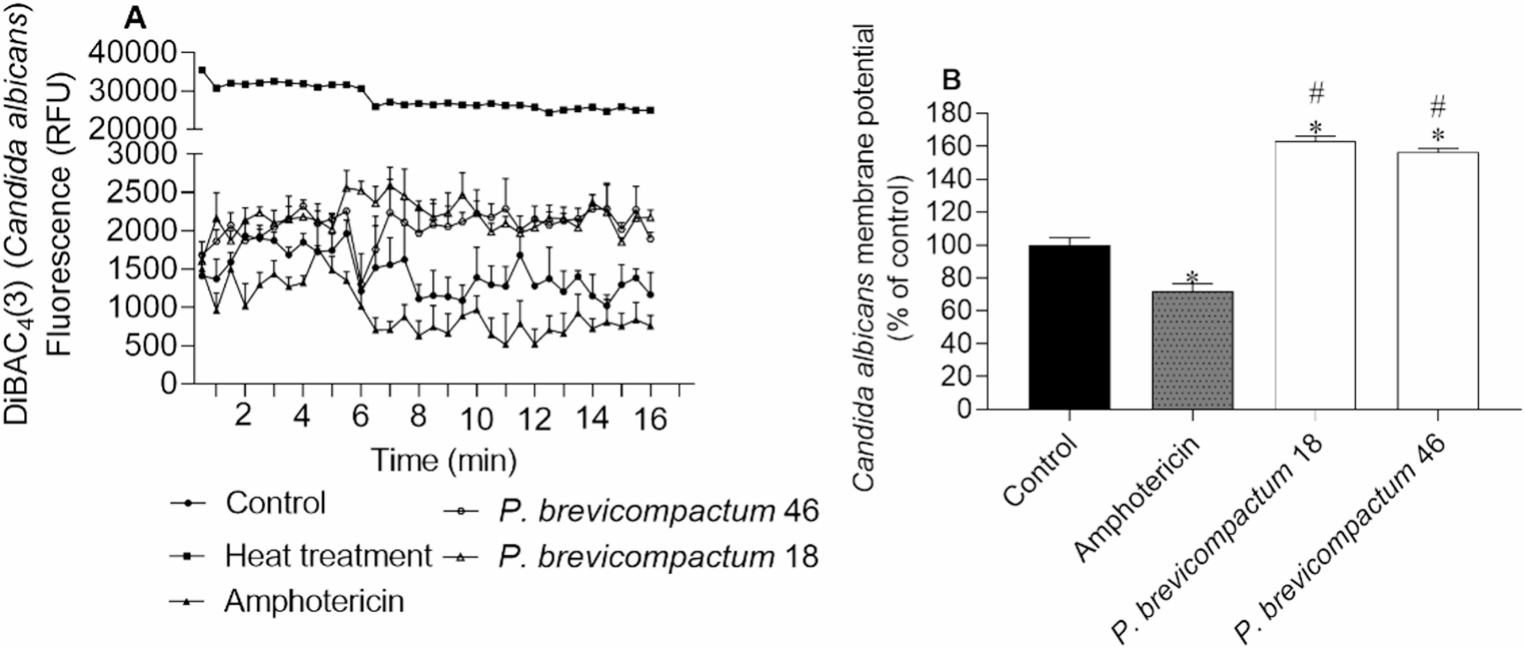
*Candida albicans* membrane potential when exposed to extracts (IC_50_ values of 19.5 and 47.8 µg/mL for extracts of isolates 18 and 46, respectively), labelled with DiBAC_4_(3) probe; **(A)** 30 s interval readings for 10 min; **(B)** after 10 min. DMSO was used as a negative control and amphotericin (4 µg/mL) as a positive control of membrane potential alterations. The values correspond to the mean ± SEM of three independent experiments. Symbols represent significant differences (One-Way ANOVA, Dunnett’s test; *p* < 0.05) when compared to the control (*) and amphotericin (#)

The effects of bioactive extracts on the membrane potential of *S. aureus*,* E. coli*,* P. aeruginosa* and *C. albicans* are demonstrated in Figs. 2, 3, 4 and 5, respectively.

Changes in membrane potential determined by the DiBAC_4_(3) method (Figs. 2 and 3) suggest that extracts of isolates 14 and 81 of *P. rubens* induced membrane depolarization in *S. aureus* and *E. coli*, as indicated by the higher fluorescence emission compared to the control.

The extract of isolate 80 of *P. rubens* promoted membrane hyperpolarization in *S. aureus*,* E. coli* and *P. aeruginosa*, exhibiting lower fluorescence emission when compared to the control. The extract of isolate 14 also promoted hyperpolarization in the *P. aeruginosa* membrane, although the effect was not so pronounced when compared to the positive control, FCCP (Fig. 4).

On the other hand, extracts of isolates 18 and 46 of *P. brevicompactum* promoted *C. albicans* membrane depolarization, suggested by the higher fluorescence emission when compared to the control, whereas the positive control, amphotericin, promoted membrane hyperpolarization (Fig. 5).

#### DNA Damaging Capacity

**Fig. 6:**
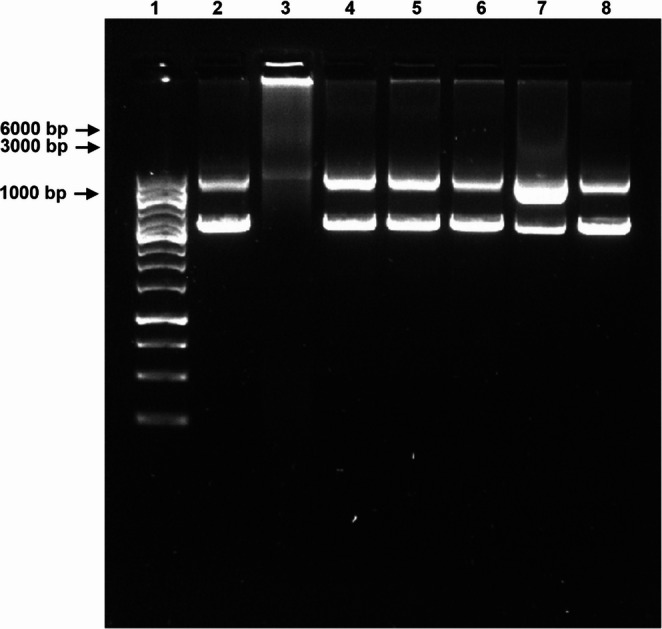
Electrophoresis gel (0.8% agarose) loaded with the DNA exposed to DMSO (negative control), ciprofloxacin, and extracts (200 µg/mL). **1 –** Marker; **2 –** Negative control (DMSO); **3 –** Positive control (ciprofloxacin, 10 µg/mL); **4 –** *P. rubens* 14; **5 –** *P. brevicompactum* 18; **6 –** *P. brevicompactum* 46; **7 –** *P. rubens* 80; **8 –** *P. rubens* 81. Electrophoresis was run at 85 V, for 45 min. The image was obtained through a gel imaging system (Gel doc). This image is representative of three independent experiments

Plasmidic DNA was exposed to the most active extracts, and possible fragmentation was verified by gel electrophoresis (Fig. 6).

The presence of a smear of the DNA bars, as well as lower DNA intensity in the supercoiled form, when compared to the control (DMSO), can be suggestive of DNA fragmentation. Ciprofloxacin promoted extensive DNA damage (lane 3). All the tested extracts revealed the ability to damage the plasmid DNA (Fig. 6), but isolate 80 of *P. rubens* exhibited the most intense smear, suggesting higher damage.

### Chemical Screening of the Active Fungal Extracts

All bioactive extracts were chemically analysed by LC-Q-TOF MS, except for the one produced by isolate 81. This was because isolates 80 and 81 were recovered from the same host, location, and sampling period, and their extracts exhibited similar results in most of the previous tests, strongly suggesting that both isolates belong to the same fungal strain.

In the chemical analyses performed, only compounds with a similarity score higher than 90% were considered (Supplementary Tables 2, 3, 4 and 5). Most compounds were nitrogenated, although some fluorinated and sulfated molecules were also present in the extracts. A comparison of the chemical profiles of extracts obtained from the strains of the same species revealed that, although they shared most compounds (peptides, amino acids and amino acid derivates), there were also unique ones.

## Discussion

### Marine Endophytic Fungi Associated with Different Seaweed Host Species

The results obtained in this study corroborate the current knowledge that the majority of marine fungal species associated with seaweeds belonged to the Ascomycota phyla, and are included in Sordariomycetes, Dothideomycetes and Eurotiomycetes (Zuccaro et al. 2008; Jones et al. 2009; Suryanarayanan et al. 2012; Hong et al. 2015; Gnavi et al. 2016, 2017; Vallet et al. 2018; Garzoli et al. 2018; Lee et al. 2019a; Wainwright et al. 2019; Poli et al. 2022).

Twenty-six out of the 27 fungi identified to the species level have previously been reported from marine habitats (Table 2), where they occur as saprotrophs, parasites, or symbionts/endobionts.

**Table 2:** Group of seaweeds based on colour from which the endophytic fungal species isolated in this study were recovered before as epiphytes or endophytes (B – brown; R – red; G – green; NM – non-mentioned), and other hosts and substrates worldwide from which the same fungal species were retrieved

Identified fungal taxa (Seaweed host)	References in marine environments					
	Seaweeds				Other hosts	Other substrates
	B	*R*	G	NM		
***Cladosporium allicinum*** (B)	[1,2]	-	[3]	-	[4–6]	-
***Cladosporium ramotenellum*** (R)	[1,7]	[8]	-	[9]	[10]	[10–12]
***Cladosporium austrohemisphaericum*** (R)	-	-	-	-	-	-
***Periconia byssoides*** (B)	-	[13]	[13]	-	[14]	-
***Stemphylium lycopersici*** (B)	-	-	-	-	[15]	-
***Stemphylium vesicarium*** (B)	[2]	-	-	-	[2]	[12,16]
***Emericellopsis maritima*** (R, B)	-	[17]	-	-	[18,19]	[10, 20–23]
***Yunnania carbonaria*** (R)	-	-	-	-	-	[24]
***Lindra obtusa*** (R)	[25]	-	-	-	-	-
***Apiospora marii*** (B)	-	-	[3]	-	-	[26]
***Exophiala mesophila*** (B)	-	-	-	-	-	[27]
***Aspergillus pseudoglaucus*** (R)	[28,29]	[29]	-	-	[30–33]	[29, 30–35]
***Aspergillus subalbidus*** (B)	-	-	-	-	-	[29]
***Aspergillus fumigatus*** (G)	[1,29,36,37]	[29]	[38]	-	[4,19,29,31,39–43]	[20,29,34,44–48]
***Aspergillus conicus*** (R)	[1,28]	[8]	-	-	[31]	[10,49]
***Aspergillus austroafricanus*** (B)	-	-	-	-	-	[50]
***Aspergillus protuberus*** (B, R, G)	-	[51]	-	-	[52,53]	[54–56]
***Aspergillus sydowii*** (B)	[29,57]	[29,57–59]	[29,57]	-	[29,31,52,60–66]	[29,34,45,49,67–76]
***Penicillium glabrum*** (R)	[77]	[8]	-	[9]	[31,62]	[78,79]
***Penicillium spinulosum*** (B)	[1]	[28]	[59]	-	[4,41]	[79,80]
***Penicillium brevicompactum*** (B)	[1,7,81]	[8,82]	[3]	-	[4,19,31,41,42,52,62,78,83–88]	[12,45,73,76,78,79,89–91]
***Penicillium antarcticum*** (B)	[1,36,92]	-	[3]	-	[4,10,19,31,33,52,78,93]	[10,45,78,94]
***Penicillium rubens*** (B, R)	-	[57]	-	[95]	[33,42,78,86,96]	[42,76,78,97,98]
***Penicillium citrinum*** (B, R)	[1,28,36]	[57,99]	[51,57]	-	[52,62,65,78,100–107]	[45,47, 56, 71,76,79,104,108,109]
***Botrytis cinerea*** (B)	[25,110]	[59]	[3]	[9]	[4,19,42,84]	[45,111]
***Asteromyces cruciatus*** (B, R)	[25,36,112]	-	-	-	[113,114]	[115–117]
***Geotrichum candidum*** (B)	-	-	[65]	-	[65]	[118]

(1) Garzoli et al. 2018; (2) Pasqualetti et al. 2020; (3) Gnavi et al. 2017; (4) Bovio et al. 2019; (5) Liu et al. 2020; (6) Poli et al. 2022; (7) Patyshakuliyeva et al. 2020; (8) Le Strat et al. 2025; (9) Cooper et al. 2022; (10) Marchese et al. 2021; 11) Li and Li, 2014; 12) Villanueva-Silva et al. 2021; 13) Sahoo et al. 2021; 14) Numata et al. 1997; 15) Li et al. 2020; 16) Azevedo et al. 2023; 17) Kannan et al. 2024; 18) Furtado et al. 2019; 19) Bovio et al. 2018; 20) Overy et al. 2014; 21) Tibell et al. 2020; 22) Perazzoli et al. 2023; 23) Virués-Segovia et al. 2023; 24) González et al. 2000; 25) Zuccaro et al. 2008; 26) Shi et al. 2021; 27) Cheng et al. 2023; 28) Godinho et al. 2013; 29) Lee et al. 2023; 30) Smetanina et al. 2007; 31) Poli et al. 2020; 32) Peng et al. 2023; 33) Borzykh et al. 2022; 34) Lee et al. 2016; 35) Gonçalves et al. 2017; 36) Lee et al. 2019; 37) Flewelling et al. 2013; 38) Li et al. 2013; 39) Yamada et al. 2007; 40) Wang et al. 2008; 41) Panno et al. 2013; 42) López-Legentil et al. 2015; 43) Hassan et al. 2024; 44) Zhao et al. 2010; 45) Bovio et al. 2017; 46) Grovel et al. 2003; 47) Maciá-Vicente et al. 2008; 48) Han et al. 2020; 49) Quemener et al. 2020; 50) Li et al. 2021; 51) Furbino et al. 2014; 52) Marchese et al. 2020; 53) Kato et al. 2007; 54) Mathan et al. 2011; 55) Corral et al. 2018; 56) Garzoli et al. 2015a; 57) Sarasan et al. 2020; 58) Teuscher et al. 2006; 59) Flewelling et al. 2013; 60) Ein-Gil et al. 2009; 61) He et al. 2012; 62) Paz et al. 2010; 63) Wang et al. 2014; 64) Liu et al. 2019; 65) Liu et al. 2018; 66) de Paula and Porto 2020; 67) Wang et al. 2018; 68) De Leo et al. 2019; 69) Ren et al. 2010; 70) Chung et al. 2013; 71) Zhang et al. 2014; 72) Xu et al. 2017b; 73) Butinar et al. 2011; 74) Burgaud et al. 2009; 75) Damare et al. 2006; 76) Kis-Papo et al. 2001; 77) Zhang et al. 2009; 78) Park et al. 2014; 79) Bubnova 2010; 80) Amer and Ibrahim 2019; 81) Fan et al. 2019; 82) Atalla et al. 2011; 83) Bringmann et al. 2004; 84) Wiese et al. 2011; 85) El-Hawary et al. 2018; 86) Heydari et al. 2019; 87) Xin et al. 2007; 88) Rovirosa et al. 2006; 89) Xu et al. 2017a; 90) Park et al. 2019; 91) Matsuo et al. 2019; 92) Leshchenko et al. 2022; 93) Vansteelandt et al. 2012; 94) Yurchenko et al. 2023; 95) Venkatachalam et al. 2024; 96) Al-Rajhi et al. 2022; 97) Xu et al. 2018; 98) Wu et al. 2024; 99) Tsuda et al. 2004; 100) Yurchenko et al. 2013; 101) Kawahara et al. 2012; 102) Shaumi et al. 2021; 103) Huang et al. 2016; 104) Christophersen et al. 1999; 105) Amagata et al. 2003; 106) Woo et al. 1995; 107) Khamthong et al. 2012; 108) Anh et al. 2024; 109) Chen et al. 2022; 110) Vallet et al. 2018; 111) Niu et al. 2019; 112) Zhuravleva et al. 2022; 113) Höller et al. 1999; 114) Höller et al. 2000; 115) Sridhar et al. 2012; 116) Rama et al. 2014; 117) Igboeli et al. 2019; 118) Gad et al. 2021

Of these species, 21 were retrieved from various seaweed species. *Aspergillus subalbidus*, *Aspergillus austroafricanus*, *Cladosporium austrohemisphaericum*, *Exophiala mesophila*, *Stemphylium lycopersici*, and *Yunnania carbonaria* were reported for the first time in this study, as endophytes of seaweeds, as far as is known.

Most of these fungal species represent halotolerant/osmotolerant genera, which are commonly found in terrestrial environments and are also widespread in various marine habitats, exhibiting a broad distribution, such as *Aspergillus*, *Penicillium*, *Talaromyces* and *Cladosporium* (Nicoletti and Vinale 2018; Hou et al. 2019; Nicoletti et al. 2023).

Although the diversity of the mycobiota associated with seaweeds was not fully assessed in this study, as it was not the primary focus, the results pointed out to a dominance of the *Aspergillus* and *Penicillium* genera within the fungal communities, consistent with findings from previous similar studies, i.e., (Suryanarayanan et al. 2010; Flewelling et al. 2013b, a; Godinho et al. 2013; Gnavi et al. 2017; Garzoli et al. 2018; Fan et al. 2019; Lee et al. 2019a; Wainwright et al. 2019; Sahoo et al. 2021). However, it is also important to note that this pattern may not accurately reflect the true endophytic fungal community composition, given the biases inherent to culture-dependent approaches.

Nevertheless, the high representativeness of *Aspergillus* and *Penicillium* species in these communities, along with their occurrence across taxonomically unrelated seaweeds, suggests a broad host range rather than strict host specificity.

.

The less strict relationship with their hosts has been attributed to their high ecological plasticity, enabling them to adapt and thrive in highly challenging and variable environmental conditions (Suryanarayanan et al. 2010; Nicoletti and Vinale 2018; Poli et al. 2020; Sarasan et al. 2020). The ability of these fungi to overcome the chemical barrier of potential hosts during the colonization process has prompted Suryanarayanan et al. (2010, 2012) to suggest that they might have coevolved with the seaweeds. Moreover, the frequent recovery of *Aspergillus* and *Penicillium* species from various marine substrate matrices suggests that these fungi are also capable of adopting or transitioning to a saprobic lifestyle, reinforcing their remarkable metabolic plasticity.

**Fig. 7:**
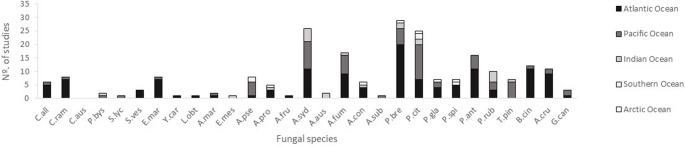
Geographic distribution of fungal species in different oceans based on the number of reports already performed

Among these genera, certain species are more commonly reported in marine environments and were also identified in this study i.e., *Aspergillus sydowii*, *Penicillium brevicompactum* and *Penicillium citrinum*. These last two species were isolated from two seaweed species, collected at two different geographical locations and periods. In addition to being recovered from a wide range of hosts and substrates, *A. sydowii*, *P. brevicompactum* and *P. citrinum*. have been documented across geographically distant locations, further highlighting their cosmopolitan nature (Fig. 7).

Even though the genus *Cladosporium* genus has been described as one of the most abundant genera in the fungal communities associated with seaweeds (Garzoli et al. 2018; Patyshakuliyeva et al. 2020; Pasqualetti et al. 2020; Vicente et al. 2021; Sahoo et al. 2021; Cooper and Walker 2022), only three different *Cladosporium* species were identified in this study, each associated with a different seaweed host and geographical location. *Cladosporium austrohemisphaericum* was identified for the first time in marine environments in this study, despite belonging to the *sphaerospermum* complex, which includes mostly halotolerant species (Zalar et al. 2007).

Similarly to these non-host specific fungi, most of the less frequent fungal species identified in this study appear to have broad ecological niches and play various ecological roles in marine environments; in fact, they are likely to occur as endobiotic partners of diverse animal and/or plant hosts or assume a saprobic mode of life in environments ranging from intertidal zones to deep-sea sediments.

In addition to these apparent generalist species, three fungal species that are considered exclusive to marine environments were also identified (Kohlmeyer and Kohlmeyer 1979; Jones et al. 2009, 2015; Overy et al. 2014; Zhuravleva et al. 2022): *Lindra obtusa*, *Emericellopsis maritima* and *Asteromyces cruciatus*.

*Lindra obtusa* was recovered in this study from a red seaweed and had previously been isolated from both healthy and decaying tissues of the brown seaweed *Fucus serratus* (Zuccaro et al. 2008). *Emericellopsis maritima* was detected on both a red and a brown seaweed and had recently been described on a red seaweed (Kannan et al. 2024). *Asteromyces cruciatus* is a widespread marine fungus found in tropical and temperate zones of the Pacific, Atlantic and Indian Oceans (Zhuravleva et al. 2022), mainly associated with marine sediments, sea foam and decaying woody substrates (Kohlmeyer and Kohlmeyer 1979; Sridhar et al. 2012; Rama et al. 2014; Igboeli et al. 2019). In this study, *A. cruciatus* was isolated from three different seaweed species on the same beach, and had been previously reported in association with living *F. serratus* (Zuccaro et al. 2008), *Agarum clathratum* (Lee et al. 2019b) and *Sargassum pallidum* (Zhuravleva et al. 2022), as well as decaying seaweeds (Zuccaro et al. 2008; Gulder et al. 2012; Sridhar et al. 2012; Tibell et al. 2020).

The absence of clear ecological patterns makes predicting the diversity of fungal communities in new or understudied hosts or habitats, more challenging. Therefore, compiling information about the hosts or substrates that fungi associate with, along with their geographical locations, is crucial for understanding and clarifying whether the occurrence of a species in a given ecological niche is random or driven by specific ecological factors.

Nevertheless, some authors have hypothesised about the way these communities are formed, based on the current research. Poli et al. (2020) proposed that the composition of the endophytic fungal communities associated with seaweed species may be defined by host-exclusive fungal species that actively select the host as a partner, as well as by generalist species that passively become trapped or adhered to the host. Garzoli et al. (2018) and Wainwright et al. (2019) suggested that the seaweed hosts themselves may play an active role in shaping and defining the fungal community, by recruiting their fungal partners from the surrounding environment.

Another important finding of this study, which requires cautious interpretation, is the higher species richness observed in brown seaweeds compared to red and green seaweeds, considering that it was not a primary objective of this study to obtain a comprehensive inventory of fungal species associated with each seaweed species. Although a similar trend has been noted in previous studies (Suryanarayanan et al. 2010; Wong et al. 2015; Zhang et al. 2016; Vallet et al. 2018; Garzoli et al. 2018; Poli et al. 2020), some authors propose intensifying the sampling effort to confirm it. This would involve collecting a larger number of green, brown, and red seaweed species over an extended period and isolating a greater number of associated fungal species.

Nevertheless, some hypotheses have been raised to explain the differences in fungal communities between groups of seaweeds. Poli et al. (2020) attributed the high diversity of the endophytic community associated with *P. pavonica* to the texture of the seaweed and, particularly, of its hairy surface that enables a higher adherence of the fungal propagules. Garzoli et al. (2015b) justified the low fungal diversity in red seaweeds with the chemical composition of the seaweeds and, specifically, the expressive production of halogenated metabolites. On the other hand, the lower diversity of the fungal communities associated with green seaweeds might be explained by the short life cycles of certain species within this group and the slow growth rate of fungal endophytes (Suryanarayanan et al. 2010).

### Antimicrobial Activity of Fungal Extracts

The antimicrobial screening tests performed with the extracts of all the fungal isolates revealed that only the strains of *P. brevicompactum* (isolates 18 and 46) and *P. rubens* (isolates 14, 80 and 81) were able to biosynthesise compounds with antimicrobial activities against *C. albicans*, and *E. coli*, *P. aeruginosa*, *S. aureus*, respectively.

The fact that two *Penicillium* species exhibited antimicrobial potential was already expected, considering that marine *Penicillium* species are recognised as major producers of a large variety of secondary metabolites with relevant biological activities, including antimicrobial properties (Flewelling et al. 2015; Singh et al. 2015; Zhang et al. 2016). According to Zhou et al. (2011) and Quemener et al. (2021), the antimicrobial activity seems to be related with the presence of genes that code for biosynthetic enzymes PKSs (polyketide synthases) and NRPSs (non-ribosomal peptide synthetases). The study of Quemener et al. (2021) revealed that every strain belonged to the *Penicillium* and *Aspergillus* genera presented at least one of these genes (two genes for *Penicillium*, and three for *Aspergillu*s, on average); genes coding for NRPS were identified in 95% of the *Penicillium* isolates, while the remaining genes were detected in ca. 40% of isolates.

The antifungal potential of *P. brevicompactum* observed in this study aligns with the findings of Heydari et al. (2019), who reported similar activity in a strain collected from a sponge. However, the considerably higher inhibitory capacity of both strains isolated in this study against a pathogenic yeast underscores their potential relevance to prospective medical applications. On the other hand, and contrasting with the present study, those authors - along with Rovirosa et al. (2006), who focused on a *P. brevicompactum* strain recovered from a different sponge - also demonstrated the antibacterial properties of the metabolites produced by this species against *S. aureus*, *E. coli*, *P. aeruginosa*, and other bacteria.

The antibacterial activity of *P. rubens* extracts reported in this study is consistent with previous findings showing that this species - like other members of the section Chrysogena - can produce unique extrolites, such as the antibiotic penicillin (Houbraken et al. 2012). Similarly, Al-Rajhi et al. (2022) demonstrated the potent antibacterial activity of extracts from a *P. rubens* strain isolated from a mangrove plant against *S. aureus*, *E. coli*, and *P. aeruginosa*. However, differing from the present study, extracts from that strain also exhibited strong antifungal activity.

The significant differences in the bioactivities of compounds produced by marine fungal strains of the same species - when associated with different hosts, habitats, and geographic locations - support the hypothesis that these species may possess distinct metabolic profiles shaped by micro- and/or macro-environmental factors.

This hypothesis is further reinforced by the findings of the present study, which revealed slight to pronounced differences among extracts produced by *P. rubens* strains collected from the same location but associated with different seaweed hosts. Specifically, the extract from isolate 95 exhibited no antimicrobial properties, whereas those from isolates 14, 80, and 81 showed slightly variations in antibacterial activity and mechanisms of action. This intraspecific variation has also been reported by Marchese et al. (2020), who emphasised the importance of intensifying bioprospecting efforts with diverse strains to unlock the biotechnological potential of fungi.

Based on this hypothesis, the absence or weak antimicrobial activity exhibited by the remaining fungal extracts screened in this study, compared with previous reports on the same species, is neither unexpected nor surprising. Concretely, the following species were recovered from different marine sources and revealed an ability to produce antimicrobial compounds against one (or more) of the tested pathogens: *C. ramotenellum* (deep-sea sediments, Villanueva-Silva et al. (2021)); *C. allicinum* (sea anemone, Liu et al. (2020)); *S. vesicarium* (deep-sea sediments, Villanueva-Silva et al. (2021)); *E. maritima* (sea foam, Overy et al. (2014); intertidal sediments, Virués-Segovia et al. (2023)); *P. lilacinum* (jellyfish, Yue et al. (2015); deep-sea sediments, Li et al. (2024)); *B. cinerea* (seaweed, Flewelling et al. (2013a)); *A. cruciatus* (seaweeds, Gulder et al. (2012), Zhuravleva et al. (2022)); *A. pseudoglaucus* (seaweed, Atalla et al. (2011)); *A. protuberus* (marine sediments, Mathan et al. (2011); seawater, Corral et al. (2018)); *A. fumigatus* (seaweeds, Flewelling et al. (2013b); sponges, Hassan et al. (2024); marine sediments, Han et al. (2020)); *A. conicus* (oceanic crust, Quemener et al. (2021)); *A. sydowii* (seaweeds, Teuscher et al. (2006), Flewelling et al. (2013a); drifwood, Zhang et al. (2008); oceanic crust, Quemener et al. (2021)); *P. glabrum* (seaweed, Zhang et al. (2009)); *P. spinulosum* (seaweed, Flewelling et al. (2013a)); *P. citrinum* (gorgonian sea fan, Khamthong et al. (2012); mangrove plant, Huang et al. (2016); marine sediments, Anh et al. (2024)); *P. antarcticum* (marine sediments, Yurchenko et al. (2023)); *T. pinophilus* (estuarine sediments, Wang et al. (2013); mangrove sediments, He et al. (2019), Muwawa et al. (2020); marine sponge, Machado et al. (2023)).

Therefore, to better identify the environmental key-factors that trigger the biosynthesis of bioactive compounds, it is essential to minimise the influence of the laboratory environment by standardising fermentation conditions and extraction protocols.

### Potential Antimicrobial Mechanisms of Action

Even though the high number of studies describing the biological activities of the extracts and compounds biosynthesised by marine endophytic fungi, very few were focused on the elucidation of the mechanisms of action related to these bioactivities (Nicoletti et al. 2023).

In the present work, three different methodologies were applied to disclose the mechanisms of action that can possibly be involved in the antimicrobial effects demonstrated by the extracts produced by strains of *P. brevicompactum* and *P. rubens*: membrane damage, membrane potential disbalance and DNA damage.

It is known that membrane damage can lead to the loss of energy production, increasing the susceptibility to antimicrobial substances and leading to cell lysis. In fact, this is the mechanism of action of several currently used antimicrobials, such as polymyxins, antimicrobial peptides, bacitracin, and others. However, none of the tested extracts have shown to induce membrane damage, suggesting that other mechanism of action should be involved.

As the cytoplasmatic membrane potential is essential for microorganisms’ survival and proliferation (Benarroch and Asally 2020), the effects of the active fungal extracts on membrane electrochemistry were also studied.

The active fungal extracts of *P. rubens* and *P*. *brevicompactum* induced both depolarization or hyperpolarization, depending on the target microorganism.

Membrane depolarization has been associated with bactericidal and fungicidal effects (Clementi et al. 2014; Molina Hernandez et al. 2023). In this particular situation, it is very interesting to note that the depolarization promoted by extracts was not induced by membrane rupture, as suggested by the Sytox green method and confirmed with the DIBAC method, due to the low fluorescence emission in the PI wavelength. On the other hand, bacterial membrane hyperpolarization can lead to cell death through a combination of disrupted energy production, impaired transport processes, loss of membrane integrity and disruption of metabolic pathways (Lee et al. 2019a; Benarroch and Asally 2020). These effects collectively contribute to the collapse of essential cellular functions and ultimately result in microbial death. Daptomycin, a lipopeptide antibiotic, promotes depolarization and disruption of membrane potential, by interacting with membrane phospholipids and leading to the release of ions from the cytoplasm into the extracellular matrix (Kırmusaoğlu et al. 2019). This mechanism is also observed in *Pseudomonas aeruginosa* upon treatment with polymyxin B (Conrad and Gilleland 1981).

Marine-derived extracts have also shown the capacity to affect microbial cytoplasmatic membrane integrity, such as those produced by endophytic fungi isolated from seagrasses (Supaphon et al. 2013).

In agreement with the present results, several antimicrobial peptides obtained from different sources have shown ability to target bacterial and fungal DNA (He et al. 2010; Lei et al. 2019).

Taking all together, it can be suggested that the antimicrobial activities verified in the present work can be related with membrane potential disbalance of the impacted microorganisms, together with DNA damage. Nevertheless, to fully disclose the antimicrobial potential of the metabolites produced by *P. rubens* and *P. brevicompactum*, it is important to isolate and identify the chemical compound(s) responsible for the antimicrobial activities, having into consideration that the extracts are complex mixtures that contain different classes of compounds that can act as antagonists or synergistically to the observed effects.

## Chemical Screening of Active Fungal Extracts

An attempt was made to correlate the tentatively identified molecules by LC-Q-TOF-MS and/or their analogues with the inhibitory effects of the extracts against *C. albicans* (isolates 18 and 46 of *P. brevicompactum*), and against *E. coli*, *S. aureus* and *P. aeruginosa* (isolates 14 and 80 of *P. rubens*).

Apparently, the extracts biosynthesised by both strains of *P. brevicompactum* did not contain the compounds pointed out by some authors to explain the antimicrobial potential of other marine strains. Specifically, Rovirosa et al. (2006) isolated and identified four compounds – mycophenolic acid, tyrosol, mycophenolic methyl ester and methyl-3,5-dihydroxybenzoate – and attributed the antimicrobial activities of the extract to the first compound, given that it has been mentioned as antibiotically active. However, the extracts denoted the presence of compounds belonging to different chemical classes (Supplementary Tables 2, 3, 4 and 5) potentially responsible for their bioactivity. Among them, some of the peptides identified in the extracts of isolates 18 and 46 of *P. brevicompactum* may contribute to this activity, given that natural antifungal peptides have gained considerable attention as promising candidates for combating *Candida* spp. infections. These peptides possess broad-spectrum antimicrobial activity and specific efficacy (Freitas and Felipe 2023). The structures of numerous known antibacterial, antifungal, and antiprotozoal agents are derived from amino acid scaffolds. In most cases, the amino acid backbone is essential for antimicrobial activity, as these compounds frequently act as structural analogues of amino acid intermediates involved in various microbial biosynthetic pathways (Nowak et al. 2021). In a recent review, Perez-Rodriguez et al. (2022) described 20 antimicrobial peptides from different origins exhibiting activity against *Candida* spp. Another review on antimicrobial peptides with antifungal effects was reported by De Cesare et al. (2020), referring to their mode of action through the interaction with membranes and other mechanisms including cell wall inhibition and nucleic acid binding. Additionally, Flores-Holguín et al. (2019) have applied computational peptidology assisted by conceptual density functional theory for analysing five new tripeptides with antifungal activity.

Moreover, the compound N-histidyl-2-aminonaphthalene identified in the extract produced by isolate 46 of *P. brevicompactum* may also be responsible for the observed antimicrobial activities. This is supported by the findings of Kouznetsov et al. (2012), which demonstrated the cytotoxic and antifungal activities of diverse α-naphthylamine derivatives, such as N-(pyridinylmethyl)-naphthalen-1-amines. These compounds exhibited activity (MIC 25–32 µg/mL) against some human opportunistic pathogenic fungi, including *C. albicans* ATCC10231, *C. tropicalis* C131, hialohyphomycetes, and dermatophytes.

Phenols are another group of compounds with biocide capacity, including *o*-cresol identified in the extract of the isolate 46. Gallucci et al. (2014) evaluated the in vitro activity and structure-activity relationship of eleven natural phenolic compounds against fluconazole-resistant *Candida* species (*C. albicans*, *C. krusei*, *C. tropicalis* and *C. dubliniensis*) and, based on the descriptors provided by this QSAR study, the antifungal activity of ortho-substituted phenols occurs by more than one mechanism of action.

Alkaloids are also reported to have a broad range of biological properties. Sulaiman et al. (2022) reviewed the distribution, mechanisms of action, structure-activity, and antimicrobial properties of alkaloids from Asian angiosperms, concluding that most active alkaloids were mainly planar, amphiphilic, having a molecular mass between 200 and 400 g/mol. In particular, pancratistatin, identified in the extracts of isolates 18 and 46, is an alkaloid found in some plant species, e.g., *Amaryllis* sp. However, other alkaloids with a similar structure such as (−) amarbellisine and (+)-hippeastrine have shown antifungal activity against *Candida albicans* (Evidente et al. 2004).

The compound 2-phthalimidoglutaric acid was also identified in both fungal extracts. Natural products containing a phthalimidine core exhibit a broad range of bioactivities and are one of the most promising targets for discovering new drug candidates. This group of molecules is mainly isolated from fungi, but also from plants or bacteria.

Although the extracts of isolates 18 and 46 had a similar chemical composition, differences in their antifungal activity against *C. albicans* may be attributable to molecules absent from the extract of isolate 46, such as lansiumarin A, 2-methyl-1-nitroanthraquinone, and 3-(4-morpholinopyrido[3’,2’:4,5]furo[3,2-d]pyrimidin-2-yl)phenol (PI-103) (Supplementary Tables 2 and 3). Lansiumarin A is a member of psoralens already reported to have antifungal bioactivity at 100 µg/mL (Yu et al. 2017), while 2-methyl-1-nitroanthraquinone belongs to the group of anthraquinones and analogues, which comprise a panoply of bioactivities including phytotoxic, antibacterial, antifungal, antiviral, algicide, enzyme inhibiting, cytotoxic, etc. (Masi and Evidente 2020). Finally, the compound 3-(4-morpholinopyrido [3’,2’:4,5]furo[3,2-d]pyrimidin-2-yl)phenol (PI-103) is a phosphatidylinositol 3-kinase inhibitor, a mTOR inhibitor and an antineoplastic agent. The TORC1 inhibitor rapamycin, which acts on the highly conserved TOR kinase domain, is effective in eliminating fungal pathogens such as *C. albicans* (Qi et al. 2022) suggesting a similar antifungal mechanism for PI-103 detected in the extract of isolate 18 of *P. brevicompactum*.

Interestingly, in the present study, extracts with antifungal activity against *C. albicans* had no antibacterial effect against *E. coli*, *K. pneumoniae*, *S. aureus*, and *P. aeruginosa* (IC_50_ values > 200 µg/mL), and vice-versa, i.e., extracts of isolates 14, 80 and 81 of *P. rubens* had no effect against *C. albicans* (IC_50_ values > 200 µg/mL). However, these last extracts demonstrated antibacterial activity over the studied bacterial strains, excepting *K. pneumoniae* (IC_50_ values > 200 µg/mL).

The extract of isolate 80 of *P. rubens* was more effective against *E. coli* (IC_50_ =3.2 µg/mL) and *P. aeruginosa* (IC_50_ =1.2 µg/mL) when compared with the extract of isolate 14 (IC_50_ =8.9 µg/mL and IC_50_ =104.0 µg/mL, respectively). This last one was more effective against the Gram-positive bacteria *S. aureus* (IC_50_ =4.7 µg/mL) than isolate 80 (IC_50_ =13.2 µg/mL), which can be explained by the different chemical profiles of each extract.

The preliminary chemical analyses performed with both extracts did not detect any of the compounds identified in previous studies. Concretely, Yan et al. (2012) and Al-Rajhi et al. (2022) attributed the antimicrobial potential of marine strains *P. rubens* to the presence of an anthraquinone derivate 9-dehydroxyeurotinone, and of methyl stearate, hexadecanoic acid methyl ester, N-(4,6-dimethyl-2-pyrimidinyl)−4-(4-nitrobenzylideneamino) benzenesulfonamide and 1,2-benzenedicarboxylic acid, respectively. Nevertheless, the presence of other compounds in the extracts from isolates 14 and 80 may also contribute to their antibacterial activities.

L-norvaline and formylphosphonate were identified in both *P. rubens* extracts. Considering that L-norvaline is a non-proteinogenic amino acid (NPAA), and that certain NPAAs have been shown to exhibit antimicrobial and herbicidal activities by mimicking proteinogenic amino acids in protein synthesis or other metabolic processes, it is also possible that this compound acts through a similar mechanism (Samardzic and Rodgers 2019).

Concerning formylphosphonate, several natural phosphonate substances with antibiotic properties have been identified (Ju et al. 2014). Phosphonate-based natural antibiotics include fosfomycin, an FDA-approved antibiotic used to treat uncomplicated urinary tract infections.

Compounds such as 18-fluoro-9E-octadecenoic acid, 2-(4-methoxyphenyl) naphthalic anhydride, 1,3-propane sultone, isoamyl *p*-anisate, lentiginosine, phenylenediamine, and methylpyrazine were detected in the extract of isolate 14 of *P. rubens*. Molecules with similar structures to the above-described compounds were already reported for their antimicrobial potential. For example, 7,10-dihydroxy-8(E)-octadecenoic acid was reported for its strong antibacterial effect against several Gram-positive and Gram-negative strains with MIC values in the range of 125–1000 µg/mL (Sohn et al. 2013). Additionally, a series of novel naphthalimide aminothiazoles were developed and evaluated for their antimicrobial activity against MRSA and *E. coli*, exhibiting MIC values of 4 and 8 µg/mL, respectively (Chen et al. 2017). Also, the organosulfur compound 1,3-propane sultone, when combined with sulfated drugs, acts synergistically, enhancing the antibacterial effect against intestinal bacterial infections.

Isoamyl *p*-anisate in included in the class of organic compounds designated as *p*-methoxybenzoic acids and derivatives and is effective in inactivating various microorganisms, including *E. coli* (Ando et al. 2015). Moreover, novel aroylated phenylenediamine compounds enhance antimicrobial defence and maintain epithelial barrier integrity (Myszor et al. 2019). Cherniienko et al. (2022) reported the antimicrobial and odour properties of alkyl pyrazines in chocolate and cocoa products, which suggests that the 2-methylpyrazine identified in extract of the isolate 14 can be involved in the verified antibacterial capacity. Finally, lentiginosine is a dihydroxyindolizidine alkaloid only identified in the extract of isolate 14 that could explain its higher activity against *S. aureus* (IC_50_ =4.7 µg/mL). To support this hypothesis, the work of Olejníková et al. (2015) reported that some newly synthesized indolizine derivatives have shown selective toxicity to this Gram-positive bacterium.

In conclusion, this study broadens knowledge of the diversity of endophytic fungal communities associated with various seaweed hosts, the ecological patterns of fungal species, and the potential of each fugal strain to produce effective secondary metabolites against resistant pathogens. Moreover, this study focused on and, for the first time, reported the most likely mechanisms of action involved in the antimicrobial activity of the metabolites. Despite the promising results obtained with the extracts of *P. brevicompactum* and *P. rubens*, further work is required to isolate and unequivocally identify the antimicrobial compounds responsible for the observed activity, whether acting individually or synergistically. The results also emphasize the need to intensify bioprospecting efforts with different marine fungal strains of the same species to better understand species metabolism and pinpoint the key-environmental factors that stimulate bioactive compound production.

## Supplementary Information

Below is the link to the electronic supplementary material.

Supplementary Material 1 (DOCX 73.0 KB)

## Data Availability

No datasets were generated or analysed during the current study.
