# Supplementary material for: Endophytic Fungi Associated with Seaweeds as Potential Producers of Antimicrobial Compounds
Source: Mar Biotechnol (NY). 2026 May 13;28(3):82. doi: 10.1007/s10126-026-10626-1 (PMC13171950; doi:10.1007/s10126-026-10626-1)
Supplement: Supplementary file 1 — Supplementary Material 1 (DOCX 73.0 KB) [file 10126_2026_10626_MOESM1_ESM.docx]

**Supplementary Material**

**Endophytic fungi associated with seaweeds as potential producers of antimicrobial compounds**

Maria da Luz Calado, Débora Santos, Patrícia Susano, Susete Pintéus, Alice Martins, Joana Silva, Miguel A. M. Oliveira, Celso Alves, Rui Pedrosa, Patrick G. Murray, Katie Shiels, Maria Jorge Campos

**Table 1.** Accession numbers of sequences deposited in the NCBI database

| **Identified fungal taxa (isolate code)** | **NCBI accession nº.** | | | | | | |  |
| --- | --- | --- | --- | --- | --- | --- | --- | --- |
|  | **ITS** | **28S** | **18S** | **cal** | **tub** | **tef1-α** | **act** | **RPB2** |
| *Cladosporium allicinum* (10) | PX093971 | PX097025 |  |  |  |  | PX123228 |  |
| *Cladosporium ramotenellum* (41) | PX093984 |  |  |  |  |  | PX116172 |  |
| *Cladosporium austrohemisphaericum* (33) | PX093982 |  |  |  |  |  | PX123229 |  |
| *Periconia byssoides* (43) | PX093986 | PX128547 | PX097051 |  |  | PX132377 |  |  |
| *Alternaria* sp. 1 (70) | PX093995 |  |  |  |  |  |  |  |
| *Alternaria* sp. 2 (72) | PX093994 |  |  |  |  |  |  |  |
| *Stemphylium lycopersici* (49) | PX093989 |  |  | PX104365 |  |  |  |  |
| *Stemphylium vesicarium* (42) | PX093985 |  |  | PX104366 |  |  |  |  |
| *Sarocladium* sp. (77) | PX093999 | PX097026 |  |  |  |  |  |  |
| *Emericellopsis maritima* (31) | PX093980 |  |  |  | PX132372 | PX104376 |  |  |
| *Emericellopsis maritima* (76) | PX093998 |  |  |  | PX132373 | PX123230 |  |  |
| *Yunnania carbonaria* (66) | PX093991 | PX097027 |  |  | PX132374 | PX104377 |  |  |
| *Lindra obtusa* (32) | PX093981 | PX128548 | PX128550 |  |  |  |  |  |
| *Apiospora marii* (68) | PX093992 |  |  |  | PX116159 |  |  |  |
| *Exophiala mesophila* (47) | PX093988 |  |  |  |  |  |  |  |
| *Aspergillus pseudoglaucus* (12) | PX093973 |  |  | PX104367 |  |  |  |  |
| *Aspergillus subalbidus* (75) | PX093997 |  |  | PX104368 | PX132375 |  |  |  |
| *Aspergillus fumigatus* (28) | PX093979 |  |  | PX104369 |  |  |  |  |
| *Aspergillus conicus* (34) | PX093983 |  |  | PX104370 |  |  |  |  |
| *Aspergillus austroafricanus* (84) | PX094003 |  |  | PX104371 | PX132376 |  |  |  |
| *Aspergillus protuberus* (3) | PX093966 |  |  | PX104372 |  |  |  |  |
| *Aspergillus protuberus* (9) | PX093970 |  |  | PX104373 |  |  |  |  |
| *Aspergillus protuberus* (23) | PX093976 |  |  |  | PX116160 |  |  | PX232369 |
| *Aspergillus protuberus* (82) |  |  |  |  | PX116161 |  |  | PX232370 |
| *Aspergillus sydowii* (25) | PX093977 |  |  | PX104374 |  |  |  |  |
| *Penicillium glabrum* (7) | PX093969 |  |  |  | PX116162 |  |  |  |
| *Penicillium spinulosum* (11) | PX093972 |  |  | PX104375 |  |  |  |  |
| *Penicillium brevicompactum* (18) | PX093975 |  |  |  |  |  |  |  |
| *Penicillium brevicompactum* (46) | PX093987 |  |  |  |  |  |  | PX232371 |
| *Penicillium antarcticum* (87) | PX094004 |  |  |  | PX116163 |  |  | PX232372 |
| *Penicillium rubens* (14) | PX093974 |  |  |  | PX116164 |  |  |  |
| *Penicillium rubens* (80) | PX094001 |  |  | PX132370 | PX116165 |  |  | PX232373 |
| *Penicillium rubens* (81) | PX094002 |  |  | PX132371 | PX116166 |  |  |  |
| *Penicillium rubens* (95) | PX094006 |  |  |  | PX116167 |  |  |  |
| *Penicillium citrinum* (45) |  |  |  |  | PX116168 |  |  |  |
| *Penicillium citrinum* (78) | PX094000 |  |  |  | PX116169 |  |  |  |
| *Penicillium citrinum* (94) | PX094005 |  |  |  | PX116170 |  |  |  |
| *Talaromyces* sp. 1 (4) | PX093967 |  |  |  | PX116171 |  |  |  |
| *Botrytis cinerea* (27) | PX093978 |  |  |  |  |  |  |  |
| *Asteromyces cruciatus* (6) | PX093968 |  |  |  |  |  |  |  |
| *Asteromyces cruciatus* (65) | PX093990 |  |  |  |  |  |  |  |
| *Asteromyces cruciatus* (69) | PX093993 |  |  |  |  |  |  |  |
| *Geotrichum candidum* (73) | PX093996 |  |  |  |  |  |  |  |

**
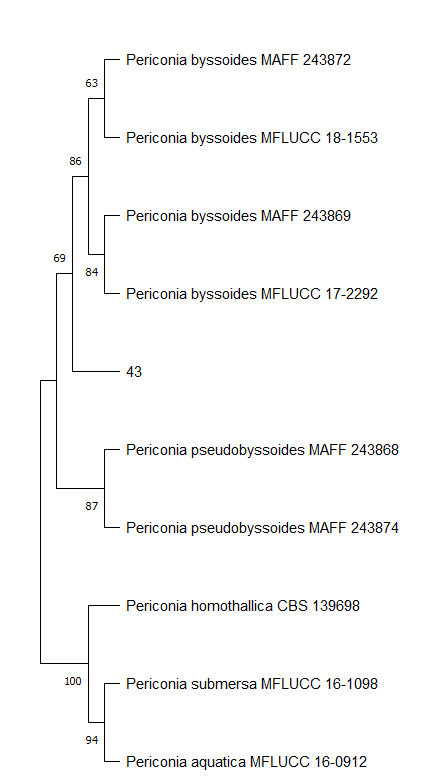
**

**Figure 1.** Maximum likelihood phylogenetic tree inferred from a concatenated dataset of ITS, 28S and tef1-α sequences of isolate 43 and BLAST best hits. ML analysis was based on the Tamura-Nei model and the tree was obtained from an initial bio-neighbour-joining tree automatically generated by the software, followed by a heuristic search using the nearest-neighbour interchange algorithm. A discrete gamma distribution was used to model evolutionary rate differences among sites. The percentage of trees in which the associated taxa clustered together is shown next to the branches.

**
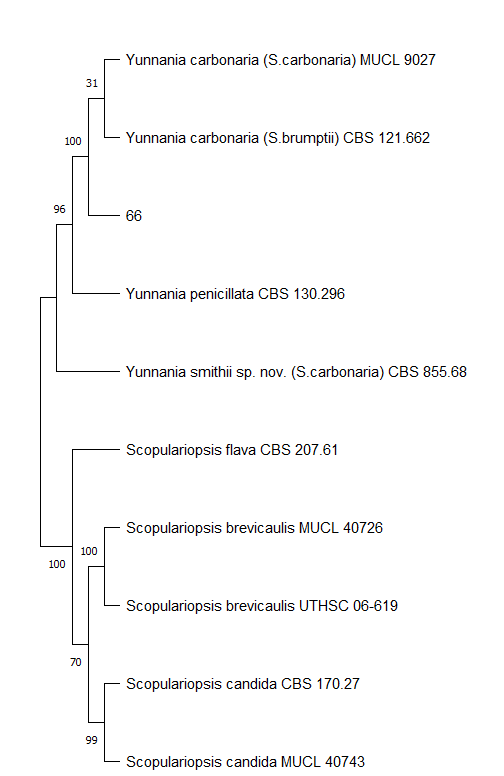
**

**Figure 2.** Maximum likelihood phylogenetic tree inferred from a concatenated dataset of ITS, 28S, tub and tef1-α sequences of isolate 66 and BLAST best hits. ML analysis was based on the Tamura-Nei model and the tree was obtained from an initial bio-neighbour-joining tree automatically generated by the software, followed by a heuristic search using the nearest-neighbour interchange algorithm. A discrete gamma distribution was used to model evolutionary rate differences among sites. The percentage of trees in which the associated taxa clustered together is shown next to the branches.

**Table 2.** Tentative LC–MS identification of compounds in extracts from isolate 18 (*P. brevicompactum*)

| Compounds | Formula | m/z | Height | Mass | RT | Score |
| --- | --- | --- | --- | --- | --- | --- |
| Hexahydro-6,7-dihydroxy-5-(hydroxymethyl)-3-(2-hydroxyphenyl)-2H-pyrano[2,3-d]oxazol-2-one | C13 H15 N O7 | 315,1188 | 649550 | 297,0849 | 24,99 | 99,92 |
| Tyr Asp Trp | C24 H26 N4 O7 | 500,2141 | 797245 | 482,1802 | 24,74 | 99,81 |
| 5-Hydroxy-6-methoxyindole glucuronide | C15 H17 N O8 | 357,1294 | 1E+06 | 339,0956 | 27,21 | 99,79 |
| Ser-Phe | C12 H16 N2 O4 | 275,1001 | 1E+06 | 252,1108 | 27,19 | 99,78 |
| Thr-Ser-OH | C13 H16 N2 O8 | 329,098 | 471474 | 328,0908 | 24,47 | 99,75 |
| Lansiumarin A | C21 H20 O5 | 353,1381 | 90367 | 352,1309 | 24,42 | 99,55 |
| Ser-Ser-OH | C12 H14 N2 O8 | 315,0824 | 1E+06 | 314,0752 | 22,41 | 99,55 |
| PI-103 | C19 H16 N4 O3 | 366,1558 | 346955 | 348,122 | 21,04 | 99,54 |
| N4-Acetylcytidine | C11 H15 N3 O6 | 286,1034 | 849596 | 285,0961 | 27,04 | 99,53 |
| Pancratistatin | C14 H15 N O8 | 343,1136 | 2E+06 | 325,0798 | 24,17 | 99,5 |
| Trp Trp Val | C27 H31 N5 O4 | 507,2713 | 83696 | 489,2375 | 33,68 | 99,49 |
| Asn Asn Gly | C10 H17 N5 O6 | 304,1248 | 75210 | 303,1176 | 24,34 | 99,48 |
| Allopurinol-1-ribonucleoside | C10 H12 N4 O5 | 286,1144 | 53407 | 268,0806 | 27,23 | 99,46 |
| 2-Methyl-1-nitroanthraquinone | C15 H9 N O4 | 285,0872 | 306226 | 267,0534 | 23,89 | 99,44 |
| 2,8-Dihydroxyquinoline-beta-D-glucuronide | C15 H15 N O8 | 355,1137 | 356478 | 337,08 | 25,33 | 99,37 |
| Leu-Asp-OH | C15 H18 N2 O8 | 355,1137 | 356183 | 354,1065 | 25,33 | 99,37 |

**Table 3.** Tentative LC–MS identification of compounds in extracts from isolate 46 (*P. brevicompactum*)

| Compounds | Formula | m/z | Height | Mass | RT | Score |
| --- | --- | --- | --- | --- | --- | --- |
| Trifluoroacetic acid | C2 H F3 O2 | 112,9856 | 139441 | 113,9929 | 24,034 | 99,96 |
| Phosphoglycolic acid | C2 H5 O6 P | 154,9751 | 18501 | 155,9824 | 18,903 | 99,88 |
| Formylphosphonate | C H3 O4 P | 154,9751 | 18047 | 109,9769 | 18,903 | 99,88 |
| *O*-Cresol | C7 H8 O | 107,0501 | 63644 | 108,0573 | 18,933 | 99,71 |
| Thr-Thr-OH | C14 H18 N2 O8 | 365,0956 | 2408121 | 342,1066 | 23,894 | 99,65 |
| N-Histidyl-2-Aminonaphthalene (βNA) | C16 H16 N4 O | 303,1213 | 209783 | 280,1322 | 23,712 | 99,42 |
| Pancratistatin | C14 H15 N O8 | 343,1139 | 2490256 | 325,08 | 23,761 | 99,39 |
| m-Aminophenol | C6 H7 N O | 110,06 | 46423 | 109,0527 | 2,833 | 99,38 |
| L-phenylalanyl-L-proline | C14 H18 N2 O3 | 285,1212 | 6938 | 262,132 | 16,148 | 99,34 |
| Palmityl Trifluoromethyl Ketone | C17 H31 F3 O | 367,2468 | 65903 | 308,2328 | 24,246 | 99,33 |
| Asn Asn Gly | C10 H17 N5 O6 | 304,1249 | 1853260 | 303,1176 | 23,662 | 99,27 |
| Glu-P-2 | C10 H8 N4 | 207,0643 | 25051 | 184,0751 | 23,894 | 99,1 |
| 4-(*O*-Carboxybenzamido)glutaramic acid | C13 H14 N2 O6 | 295,0927 | 82028 | 294,0853 | 18,654 | 99,08 |
| Leu Gln Asn | C15 H27 N5 O6 | 391,2297 | 46078 | 373,1959 | 24,125 | 99,01 |
| 2-Phthalimidoglutaric acid | C13 H11 N O6 | 295,0925 | 2493029 | 277,0587 | 18,619 | 99 |

**Table 4.** Tentative LC–MS identification of compounds in extracts from isolate 14 (*P. rubens*)

| Compounds | Formula | m/z | Height | Mass | RT | Score |
| --- | --- | --- | --- | --- | --- | --- |
| Trp Thr Pro | C20 H26 N4 O5 | 403,1971 | 2E+06 | 402,1899 | 19,912 | 99,36 |
| m-Aminophenol | C6 H7 N O | 108,0454 | 102223 | 109,0527 | 16,593 | 99,95 |
| 15-Tetracosenal | C24 H46 O | 368,3889 | 82740 | 350,3551 | 33,626 | 99,78 |
| 3-Buten-1-amine | C4 H9 N | 116,0717 | 76163 | 71,0735 | 20,143 | 99,98 |
|  |  |  |  |  |  |  |
| L-Norvaline | C5 H11 N O2 | 116,0717 | 73610 | 117,079 | 20,073 | 99,96 |
| 18-Fluoro-9E-octadecenoic acid | C18 H33 F O2 | 318,28 | 62104 | 300,2461 | 23,31 | 99,32 |
|  |  |  |  |  |  |  |
| Trp Asn His | C21 H25 N7 O5 | 456,1986 | 49864 | 455,1915 | 19,862 | 99,07 |
| 2-(4-Methoxyphenyl)naphthalic anhydride | C19 H12 O4 | 322,1076 | 42694 | 304,0736 | 20,509 | 99,36 |
| Formylphosphonate | C H3 O4 P | 168,9904 | 41181 | 109,9762 | 35,484 | 99,2 |
| Aliarin | C22 H24 O8 | 434,181 | 35801 | 416,1472 | 19,846 | 99,18 |
| Trifluoroacetic acid | C2 H F3 O2 | 112,9856 | 26907 | 113,9929 | 36,346 | 99,99 |
| (S)-3-Mercaptohexyl acetate | C8 H16 O2 S | 177,0944 | 26026 | 176,0872 | 35,5 | 99,23 |
| 4-Octen-3-one | C8 H14 O | 125,0973 | 9579 | 126,1047 | 19,508 | 99,32 |
| 1,3-Propane sultone | C3 H6 O3 S | 120,9965 | 8440 | 122,0037 | 31,132 | 99,13 |
| 3-Butyn-1-al | C4 H4 O | 127,0402 | 5559 | 68,0263 | 21,725 | 99,51 |
| (S)-Ureidoglycolic acid | C3 H6 N2 O4 | 152,0668 | 4930 | 134,033 | 25,069 | 99,38 |
| Isoamyl p-anisate | C13 H18 O3 | 221,1183 | 4232 | 222,1255 | 24,203 | 99,22 |
| 4-Isopropylphenylacetaldehyde | C11 H14 O | 221,1184 | 3179 | 162,1047 | 24,097 | 99,55 |
| Lentiginosine | C8 H15 N O2 | 156,1032 | 2805 | 157,1113 | 20,83 | 99,68 |
| Phenylenediamine | C6 H8 N2 | 153,0672 | 2226 | 108,069 | 12,357 | 99,09 |
| Methylpyrazine | C5 H6 N2 | 153,0672 | 2172 | 94,0533 | 12,357 | 99,09 |

**Table 5.** Tentative LC–MS identification of compounds in extracts from isolate 80 (*P. rubens*)

| Compounds | Formula | m/z | Height | Mass | RT | Score |
| --- | --- | --- | --- | --- | --- | --- |
|  |  |  |  |  |  |  |
| L-Norvaline | C5 H11 N O2 | 116,0718 | 139943 | 117,0791 | 19,937 | 99,75 |
| Lys Phe Phe | C24 H32 N4 O4 | 499,256 | 109831 | 440,2422 | 24,221 | 99,53 |
| Formylphosphonate | C H3 O4 P | 154,9753 | 64064 | 109,977 | 23,277 | 99,53 |
| Phosphoglycolic acid | C2 H5 O6 P | 154,9753 | 21595 | 155,9825 | 23,277 | 99,53 |
| Trifluoroacetic acid | C2 H F3 O2 | 112,9856 | 19048 | 113,9928 | 17,966 | 99,08 |
| Tyr Glu Asp | C18 H23 N3 O9 | 426,1504 | 9302 | 425,1432 | 19,794 | 99,05 |
